# A critical review on the role of leakages in the facemask protection against SARS‐CoV‐2 infection with consideration of vaccination and virus variants

**DOI:** 10.1111/ina.13127

**Published:** 2022-10-11

**Authors:** Jean Schmitt, Jing Wang

**Affiliations:** ^1^ Department of Civil, Environmental and Geomatic Engineering, ETH Zurich Institute of Environmental Engineering Zurich Switzerland; ^2^ Laboratory for Advanced Analytical Technologies, Empa Swiss Federal Laboratories for Materials Science and Technology Dubendorf Switzerland

**Keywords:** facemask, filtration efficiency, fit factor, infection risk, leakage, SARS‐CoV‐2

## Abstract

The protection provided by facemasks has been extensively investigated since the beginning of the SARS‐CoV‐2 outbreak, focusing mostly on the filtration efficiency of filter media for filtering face pieces (FFP), surgical masks, and cloth masks. However, faceseal leakage is a major contributor to the number of potentially infectious airborne droplets entering the respiratory system of a susceptible individual. The identification of leaking spots and the quantification of leaking flows are crucial to estimate the protection provided by facemasks. This study presents a critical review on the measurement and calculation of facemask leakages and a quantitative analysis of their role in the risk of SARS‐CoV‐2 infection. It shows that the pairing between the mask dimensions and the wearer's face is essential to improve protection efficiency, especially for FFP2 masks, and summarizes the most common leaking spots at the interface between the mask and the wearer's face. Leakage is a crucial factor in the calculation of the protection provided by facemasks and outweighs the filtration performances. The fit factors measured among mask users were summarized for different types of face protection. The reviewed data were integrated into a computational model to compare the mitigation impact of facemasks with vaccination with consideration of new variants of SARS‐CoV‐2. Combining a high adoption rate of facemasks and a high vaccination rate is crucial to efficiently control the spread of highly infectious variants.

**Practical implications**

- The literature review provides an overview of the major regulations on facemasks, the measurement and simulation methods developed to locate and quantify air and particles leakage, and their integration within larger‐scale epidemiological models.
- The presented computational model is a tool to estimate the impact of various parameters on the infection risk and helps inform decisions on mask mandates and future regulations on facemasks.
- The model focuses on the impact of the leaking and filtration properties on the infection risk and can be further adapted to consider other airborne viruses and SARS‐CoV‐2 variants.
- Future developments on facemasks need to focus on improving the fit between the mask and the wearer's head.

## 1 INTRODUCTION

Facemasks gained an explosive news coverage in early 2020 at the beginning of the COVID‐19 pandemic as a measure to mitigate the spread of the infection, and since the middle of 2020, mask recommendations and mandates have been regularly updated and modulated to adapt to the successive waves of infections.^1^, ^2^ Mask mandates were reintroduced at the end of 2021 in a number of countries amid the emergence of the latest SARS‐CoV‐2 Omicron variant^3^, ^4^, ^5^ with early reports suggesting a potentially higher infectivity compared with previous variants and a reduction of the protection provided by vaccines.^6^, ^7^, ^8^ Consequently, governments strongly advised or imposed the use of the highly efficient FFP2 (filtering face piece 2) masks in various indoor and outdoor settings.^9^, ^10^

The role of mask wearing in slowing the spread of COVID‐19 has been heavily investigated. Chu et al.^11^ published a meta‐analysis to assess the efficiency of social distancing and mask wearing, concluding that medical or surgical masks might result in a large reduction in virus infection, with N95 masks leading to a larger reduction than surgical masks. The findings on the efficiency of masks have been supported by Brooks and Butler^12^ focusing on specific indoor settings (a hair salon, a warship). From a community perspective, a high rate of mask wearing has been found to significantly reduce the reproduction number.^13^ However, Bartoszko et al.^14^ and Haller et al.^15^ pointed out the lack of evidence to conclude that N95 masks provide a higher level of protection than medical masks.

An accurate estimation of the protection provided by facemasks is challenging as it requires not only information on the filtration efficiency as a function of the particle sizes, but also knowledge of the fit between the mask and the wearer's face. Faceseal leakage plays a significant role in estimating the inhalation of infected respiratory droplets leading to the risk of SARS‐CoV‐2 infection. Leakage depends on numerous parameters such as the geometry of the mask, its resistance to the inhaled or exhaled airflow, the skin's roughness, or the relative size of the mask and the wearer's head.

The present critical review focuses on the measurement, visualization, and modeling of the faceseal leaks, complemented by the application of the gathered data to estimate the impact of leakage on the risk of SARS‐CoV‐2 infection. The level of protection provided by facemasks depends on many parameters, including but not limited to the material's filtration efficiency, the ability of the wearer to correctly fit the mask, the parameters of the interaction between an infected individual and a susceptible individual (duration, distance, ventilation). In order to build our model and estimate the impact of these parameters on the infection risk, and therefore, on the efficiency of facemasks, we mostly focused our review on laboratory experiments and theoretical considerations. Larger‐scale studies on the impact of facemask on the spread of the disease within a large population were not considered in the review. First, we summarized the leakage requirements in European standards used to regulate the protection provided by the most common types of masks (filtering face pieces [FFP], medical masks, and community masks). Then, we discussed the adoption of the fit and protection factors as an indicator of the protection provided by masks used for source control and respiratory protection, including the impact on the fit by head movements and respiratory activities, before highlighting the high variability in the fit factors of masks in realistic conditions. In the third section, we described the techniques to visualize leaks and identify the distribution of leaking spots along the contact surface between the mask and the wearer's face. In the fourth section, we reviewed numerical and analytical models developed to investigate the parameters affecting faceseal leakage, as well as the integration of the leaking fraction into physical and epidemiological models to satisfactorily predict the infection risk and the spread of the disease. Finally, we summarized the findings to define realistic levels of leakage and modeled the influence of facemasks on the risk of SARS‐CoV‐2 infection compared with the protection provided by vaccines in the face of new variants.

## 2 REGULATIONS ON THE LEVEL OF LEAKAGE

The majority of masks commonly used to reduce the transmission of SARS‐CoV‐2 can be classified into three categories: high‐efficiency face protection also called respiratory protective devices (e.g., filtering face piece 2, also known as FFP2, and N95) intended for respiratory protection; medical and surgical masks designed for source control with generally lower protection efficiencies and looser fit; community masks, defined by the Swiss National COVID‐19 Science Task Force^16^ as non‐professional masks designed to protect the public from infection through source control. These three types of face protections are regulated by various standards to ensure a minimum filtration efficiency and/or a sufficient fit quality. The respiratory protection and source control abilities of other devices, including face shields and face coverings such as clothes or scarves, are not regulated and their use is generally not encouraged.^17^

High‐efficiency protections are defined by both a minimum filtration efficiency and a maximum total inward leakage. The European standard EN 149:2001 + A1:2009 describes the requirements for FFP, divided into FFP1, FFP2, and FFP3 masks. The total inward leakage (given as the ratio of the particles concentration measured in the volume enclosed by the mask over the ambient particles concentration) is calculated from the particles penetrating through the filtering part of the mask and the particles entering through the imperfectly sealed interface between the mask and the wearer's head. As these masks are primarily intended for respiratory protection, only the inward leakage is regulated. The values for filter penetration and total inward leakage are given in Table 1. The measurement of the total inward leakage is done on volunteers performing different exercises (walking, moving the head, speaking). The EN 149 standard allows the presence of a one‐way valve designed to reduce the pressure drop at exhalation. The use of masks equipped with such a device is however highly discouraged by health authorities^17^, ^18^ in the context of the SARS‐CoV‐2 outbreak as they do not filter the released airflow, and therefore, do not protect others from emitted respiratory particles. The NIOSH‐42 CFR Part 84 is the equivalent of the EN 149 in the United States and sets the requirements for protection equipment such as N95 or N99 masks.

**TABLE 1:** Requirements for the different types of masks according to the three standards for filtering face pieces (EN 149:2001 + AC:2009), medical masks (EN 14683 + AC:2019), and community masks (CWA 17553:2020)

	EN 149:2001 + AC:2009			EN 14683 + AC:2019		CWA 17553:2020	
	FFP1	FFP2	FFP3	Type I	Type II/IIR	70%	90%
Designed for	Respiratory protection			Source control		Source control	
Filtration efficiency	80%	94%	99%	95%	98%	70%	90%
Average particles diameter for test	600 nm NaCl particles			3 μm		3 μm	
Flowrate	95 L/min			8 L/min		8 L/min	
Max. total inward leakage^a^	25%/22%	11%/8%	5%/2%	Not required		Not required	
Valve	Exhalation valves allowed			Not mentioned		Exhalation or inhalation valves prohibited	

^a^ The EN 149 standard gives two values for the total inward leakage: the first value is the limit that should not be exceeded for 46 out of 50 individuals exercise results (five exercises for each of the 10 tested individuals) and the second value is the maximum arithmetic mean for 8 out of the 10 tested individuals.

Medical (or surgical) masks are regulated by the EN 14683 + AC:2019 standard. They are designed for source control, thus preventing the spread of droplets emitted by the wearer. They are classified into two groups, Type I and Type II, based on their filtration efficiency as given in Table 1 (a third group, Type IIR, differs from Type II by the maximum allowed breathing resistance). As these masks regulate the exhaled airflow, no requirement for the inward leakage is defined in the standard. Comparable standards for medical masks include ASTM F2100‐21 defining the requirements for medical face masks labeled Level 1, Level 2, and Level 3. The ASTM F2100‐21 standard requires not only a minimum filtration efficiency at 3 μm like the EN 14683 standard, but also a minimum filtration at 0.1 μm.

Community masks are regulated by the CWA 17553:2020 standard at the European level, which was developed in 2020 following the outbreak of COVID‐19. They are primarily designed to minimize the projections of respiratory droplets (source control), but they also provide a certain degree of respiratory protection. Community masks following the CWA 17553:2020 standard are divided into two groups based on their filtration efficiency as shown in Table 1. The CWA 17553 standard highlights the importance of the fit on the wearer's face by including size requirements based on the average face morphology of the European population (adults and children). It also defines the area covered by the mask with an emphasis on the nose, cheeks, and chin where most leakages occur. Inhalation and exhalation valves are prohibited. The ASTM F3502‐21 is a similar standard adopted during the outbreak of COVID‐19 to regulate barrier face coverings in order to ensure a sufficient protection from exhaled droplets and aerosol, but also reduce the level of aerosol inhaled by the wearer.

## 3 THE FIT FACTOR AS AN INDICATOR OF THE PROTECTION PROVIDED BY MASKS

The Occupational Safety and Health Administration (OSHA) defines the fit factor as a “quantitative estimate of the fit of a particular respirator to a specific individual” which is calculated as the ratio of the aerosol concentration in the environment to the concentration in the volume enclosed by the mask and the wearer's head during a series of exercises involving movements of the head.^19^ The fit factor is measured during a fit test designed to ensure that the inward penetration of particles is below the prescribed limits in operational conditions. This metric shows a high inter‐ and intra‐user variability as it is not only dependent on the type of mask, but also on the skills and carefulness of the wearer and on the agreement between the sizes of both the mask and the wearer's head.

Originally developed to guarantee an optimal protection in a professional context, the fit factor (also called protection factor when it is not calculated within a standardized fit test) and the total inward leakage have been widely adopted to compare the protection levels of different types of masks either on volunteers^20^, ^21^, ^22^ or on manikins.^23^, ^24^, ^25^ Manikins help reducing the variability resulting from movements of the head and to a certain extend from the skills of the volunteers in adjusting the mask. They allow a rough control of the fit configuration (by means of a fully or a partially sealed interface,^24^ or via the introduction of artificial leaks).^25^, ^26^ Measurements have shown that the largest fraction of the penetrating aerosol enters via faceseal leakage rather than through the filtering part.^26^, ^27^, ^28^ The fit factor is thus a better indicator of the level of protection than the sole measurement of the filtration efficiency.

### 3.1 Measurement of the protection efficiency of masks used for source control

Measurements of the aerosol penetration was initially developed to estimate the level of respiratory protection. Assessing the protection efficiency in a source control application is more challenging as the emitted aerosol has to be distinguished from the ambient aerosol by, for example, radioactive^29^, ^30^ or fluorescent^31^ markers. The aerosol exposure can be calculated as the ratio of the radioactivity deposited on the filter of the receiving manikin over the radioactivity emitted by the source manikin, with the use of soft manikins to simulate a realistic fit.^29^, ^30^ Alternatively, the sampling apparatus can be placed directly in front of the mouth to reduce the mixing with ambient aerosol and avoid using harmful tracers.^32^ Measurements can also be performed in a closed volume with a stable ambient particles concentration,^33^ but such a setup can impact the results as a small volume might not allow a realistic spread of the emitted particles (further discussion can be found in Appendix S1). Source control was generally found to be significantly more efficient than respiratory protection at reducing the exposure to aerosol.^29^, ^30^, ^34^, ^35^ However, this relation was less pronounced and even inverted in particular settings: in small volume enclosures,^33^ or when the susceptible individual was placed next to or behind the source.^35^ A computational model has been developed to compare the efficiencies of mask usage on the emitter and the receiver with various levels of leakage.^36^ The authors did not find significant differences in the efficiency of source control versus respiratory protection. The study considered a uniform distribution of particles in the interaction volume which is valid for long‐range interactions but does not consider short‐range interactions. The main benefit of source control—a reduced velocity of the exhaled airflow and the carried particles—has therefore not been included in the model, which reduced the calculated efficiency of source control compared with respiratory protection.

### 3.2 Impact of head movements

The protection efficiency is degraded by head movements (bending over, talking, moving the head side‐to‐side and up‐to‐down, grimacing) as facial muscles modify the contact surface between the mask and the wearer,^27^, ^37^, ^38^, ^39^, ^40^, ^41^ thus creating additional leaking spots. The degradation of the protection is strongly dependent on the quality of the initial fit and the compatibility between the mask and the wearer's head. Head movements have been found to cause a lower degradation of the protection efficiency of well‐fitting masks (i.e., N95 or FFP) compared with masks providing a lower fit quality (i.e., surgical masks).^39^, ^41^ A computational framework developed to model and further investigate this aspect^42^, ^43^ is presented in the modeling section.

### 3.3 Influence of the expiratory activity

The type of expiratory activity is also likely to impact the fit: speaking has been found to degrade the respiratory protection efficiency,^37^, ^38^, ^40^, ^41^ but the impact has been partially attributed to additional particles generated by the emitter^37^ and to a measurement artifact resulting from a limited inhalation time and a longer exhalation time compared with breathing.^38^ The source control efficiency of a surgical mask has been found to be higher for speaking than for breathing.^32^ Coughing and sneezing are likely to impact the source control efficiency. On one hand, they are both violent expiratory activities and cause the airflow—and the carried particles—to be expulsed from the mouth or nose over a short period of time. This leads to high flowrates and to an increase of the pressure in the space between the mask and the wearer's head which is likely to modify the balance between the airstreams flowing through the gaps and the filtering part of the mask. On the other hand, sneezing and coughing generate larger particles^44^, ^45^ which are expulsed at a higher velocity than speaking or breathing, potentially leading to a higher fraction of the particles impacting the facemasks as they cannot follow the leaking airflow. Such particles are more likely to be filtered as the masks' filtration efficiencies increase for particle sizes above their most penetrating particle size. The capture efficiencies (not considering leaks) of N95 and surgical masks have been found to be higher upon coughing versus tidal breathing.^30^ Investigations on the outward protection upon exhaling and coughing have not highlighted significant differences between the two activities.^46^ The testing setup has been conceived to gather and measure particles from all around the emitter, including particles exiting from sideward and backward leaks. A comparison of the inward and outward protection efficiency as a function of the relative position of two manikins^35^ (front‐to‐front, front‐to‐back, and side‐to‐side) coughing and breathing has shown a significant advantage of source control over respiratory protection in the front‐to‐front and front‐to‐back orientations upon coughing, while the relation was inverted in the side‐to‐side measurements. The comparative advantage of source control appeared to be reduced for front‐to‐front and back‐to‐back for breathing, and both mitigation measures showed similar impact in the side‐to‐side configuration. A mask on the emitter therefore efficiently stops the forward motion of a cough jet but redirects a higher fraction of the particles to the leakage compared with breathing. Significant differences in the spread of emitted particles between coughing and sneezing have been measured on the side and the back of a masked source,^47^ with a sneeze leading to a larger spread of the aerosol around the emitter. The forward movement of the particles was efficiently contained, with sneezing leading to a noticeably higher spread than coughing. Measurements on a manikin featuring a pulsatile flow simulator^48^ have led to the conclusion that a succession of expiratory pulses (e.g., during a series of consecutive coughs) degrades the fitting of facemasks (higher leaking airflow) more than single isolated pulses.

### 3.4 Summary of the fit factors provided by different types of masks

**FIGURE 1:**
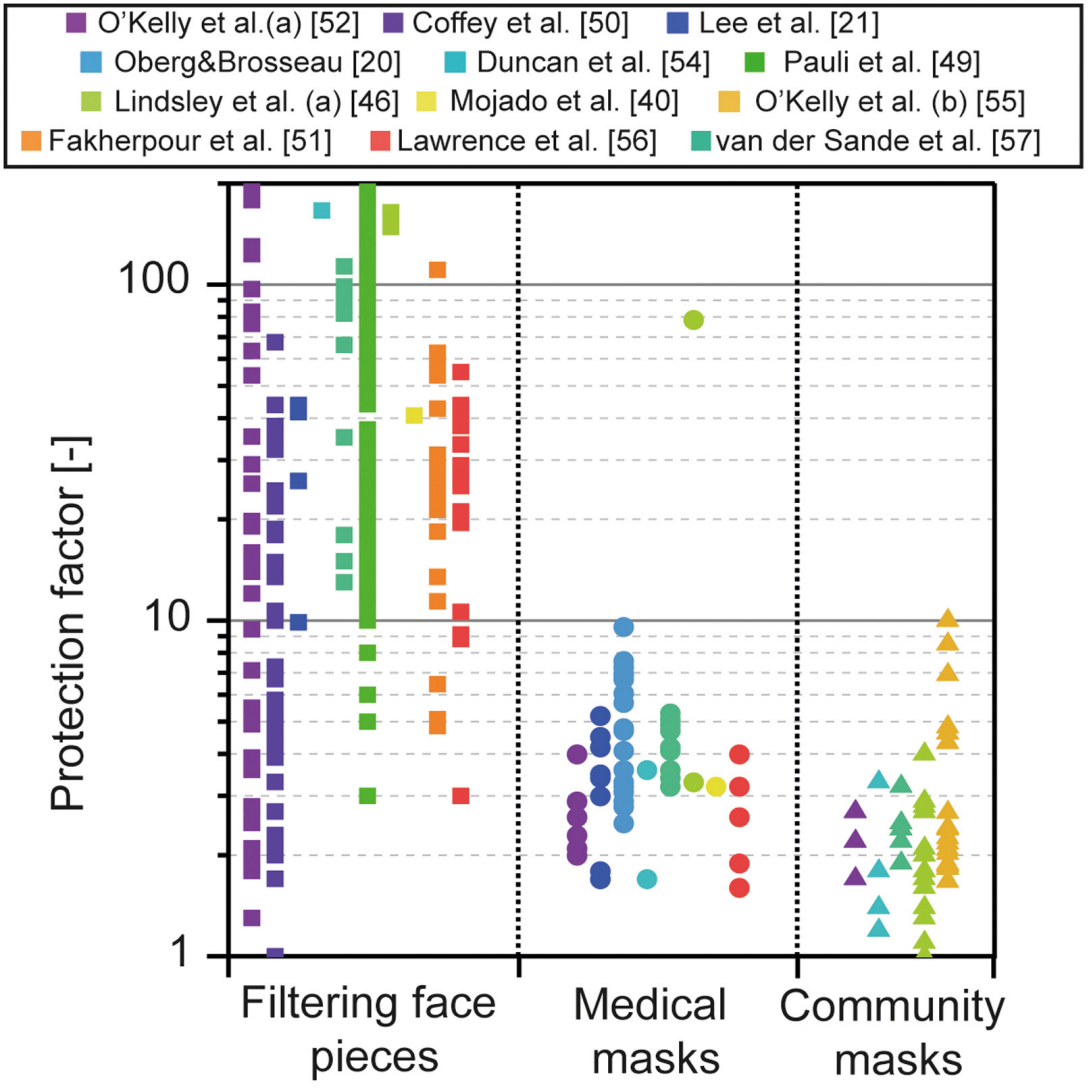
Synthesis of the measured fit factors and protection factors in the literature for various types of facemasks. The masks have been grouped into filtering face pieces, medical masks, and homemade masks. For clarity, the fit factors measured by Pauli et al. (2014)^49^ were set to 200 if measured >200. Both the fit factor and the protection factor refer to the total inward leakage (penetration through the filter and the leaks); however, the fit factor is a reserved term to quantify the performances of a mask during a standardized fit test.^54^, ^55^, ^56^, ^57^

Fit factors measured on trained and non‐trained users were summarized and organized into the three groups previously mentioned. The results are given in Figure 1 and highlight the diversity in fit qualities likely to be found within a population of users with various levels of training in mask usage. The data presented in Figure 1 is based on measurements of the total inward penetration (i.e., including the penetration through the filter and the inward leakage). The displayed fit factor refers to the ratio between the concentration outside of the mask and the concentration penetrating inside of the mask. We use the terms *fit factor* and *protection factor* to refer to the total penetration through the filter and the leaks: the fit factor is reserved to the quantification of a mask's penetration during a standardized fit test. Measurements of the fit factor on 14 experienced individuals (working in a Biosafety Level 3 laboratory)^49^ wearing 10 different FFP3 masks indicated a low success rate in the standardized fit test: only 2 out of 14 volunteers successfully passed the test on all respirators and significant disparities appeared between the masks. Comparable results have been obtained with N95 masks^50^ with an average passing rate of 16% (range 0%–76%). Low success rates obtained with a blend of FFP masks^51^ (half of the tested masks had a passing rate <10%) have been attributed to a mismatch between the facial dimensions of the wearers and the masks' sizes. Self‐assessment of mask fit by way of fit checks (feeling the leaking flow around the mask) was not correlated with the measured fit factors, as the N95 masks were highly sensitive to small leaks^52^ that could not be detected by the wearers. On the contrary, a significant improvement of the fit factor has been measured when users were allowed to adjust their N95 masks after performing a seal check.^53^ It is worth noting that a seal check is a necessary step to ensure that a certified mask (e.g., N95 or FFP) provides the intended level of protection from airborne pollutants. However, a seal check is unlikely to be performed by the general public wearing FFP or N95 masks, due to a lack of information and/or training.

## 4 METHODS DEVELOPED TO VISUALIZE THE LEAKS

Measurement of the fit factor, whether quantitative or qualitative, does not provide detailed information on the localization of the leaking spots. Various methods to visualize leaks have been implemented to provide precious information on the interaction between facemasks and the faces of the wearers: Schlieren optical technique, light scattering, thermal imaging, radioactive and fluorescent markers, and measurements of the airborne particles' concentration around the source.

### 4.1 Schlieren optical technique

**FIGURE 2:**
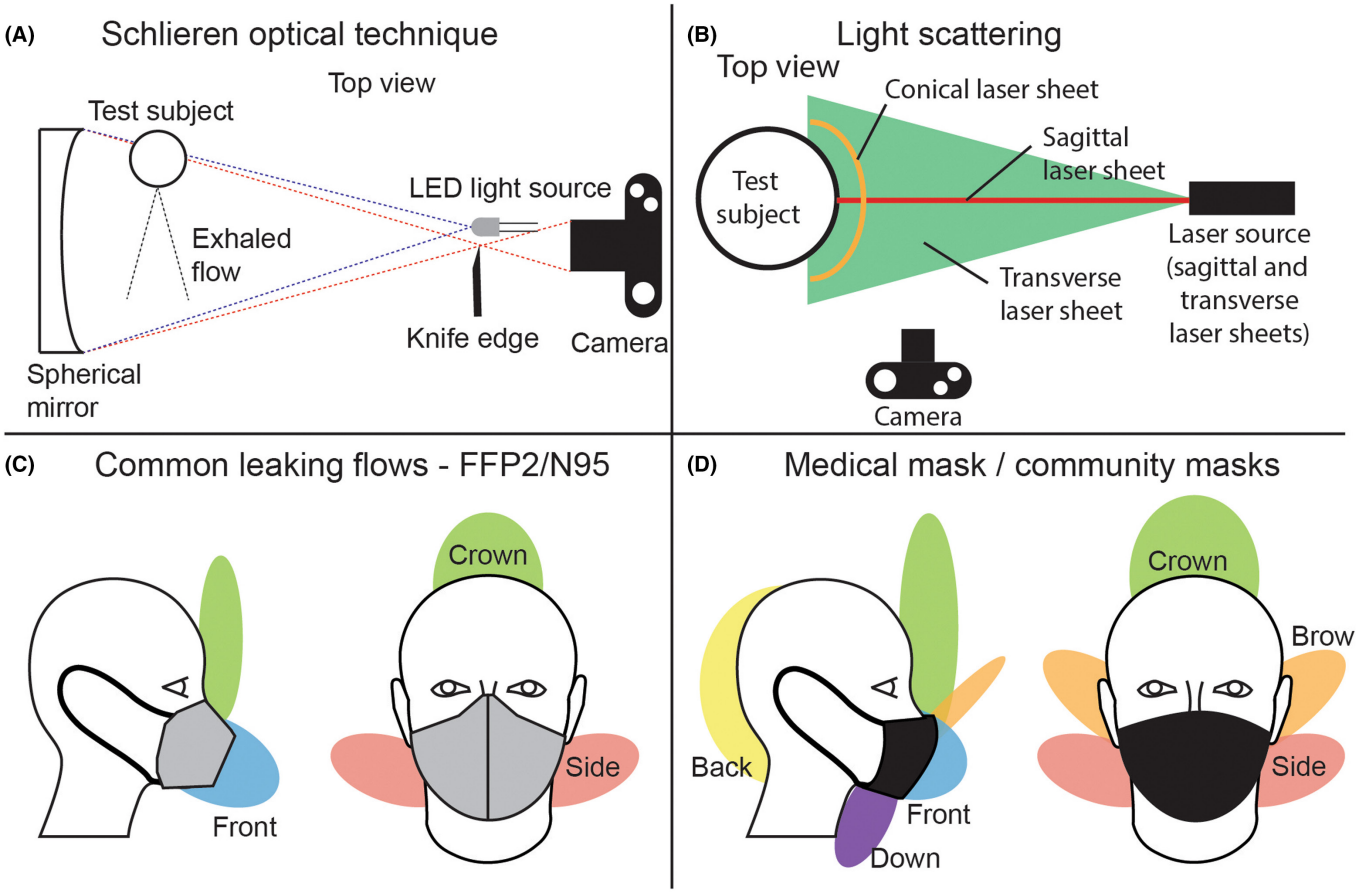
Setup similar to the one used by Tang et al.^62^ to visualize the airflow via Schlieren optical technique (A); experimental setup using light scattering to visualize the particles generated by a manikin or a volunteer (B); synthesis of the most common directions of airflows exiting facemasks as seen with Schlieren optical technique and light scattering with FFP2/N95 masks (C) and surgical/community masks (D). The nomenclature of the leaking spots is taken from Viola et al.^60^ with data taken from Viola et al.^60^ and Tang et al.^58^ The airflow through the filter media (front flow) appeared larger for N95/FFP2 masks in the Schlieren imaging Tang et al.,^58^ while laser observations showed a lower scattering intensity in the front flow compared with peripheral leaking flows, indicating a lower particles concentration as a result of filtration.

Schlieren optical technique is a powerful method to visualize the exhaled airflow. It is based on the differences in optical refraction index between the warm exhaled air and the colder surrounding made visible by a relatively simple optical setup as shown in Figure 2A. This non‐invasive technique does not require tracers to reveal the flow and can be easily performed on volunteers without additional risks. The major advantages of relying on volunteers instead of fitting masks on manikins are the qualitative illustration of the inter‐ and intrapersonal variability of the fitting, and the generation of a realistic exhalation flow which would be difficult to reproduce with a manikin. However, this technique does not provide information about the trajectories of the respiratory droplets. The filtration efficiency of masks cannot be evaluated with this technique as it would require the detection of the particles. Instead, the containment effect can be estimated through the horizontal spread of the flow and the leaking spots can be highlighted to help improving the fit. This method has been extensively used to visualize and compare the airflow patterns generated by various types of face protections,^58^, ^59^, ^60^, ^61^ as well as other interventions aimed at containing the exhaled flow.^62^ Schlieren optical technique can be combined with other visualization methods to provide quantitative or semi‐quantitative results to compare the efficiency of various types of masks (i.e., radiolabeled aerosol,^63^ fog^64^).

### 4.2 Light scattering

Light‐scattering methods allow a direct observation of the particles' trajectories. Light sensors can be implemented to measure the intensity of the scattered light and derive a semi‐quantitative analysis.^65^ This method requires the utilization of tracers mostly with manikins (e.g., nebulized NaCl solution,^66^ artificial fog^67^) but also with volunteers (e.g., with smoke from e‐cigarettes^68^). The exhaled airflow can also be visualized in a room filled with tracers prior to inhalation or exhalation.^69^

The size and position of the area covered by the laser is a critical factor as a fraction of the emitted flow might be outside of the targeted area. Particularly, the fast and narrow jet generated from an unmasked breath might appear dimmer than the slower plume generated with a mask.^66^ An example of experimental setup to track particles by light scattering is given in Figure 2B. A laser sheet placed in the sagittal plane of the test subject is the most common configuration.^67^, ^70^, ^71^, ^72^, ^73^, ^74^ Such a setup provides information on the forward motion of the emitted cloud of particles but does not fully illuminate the leaking flows not contained within the observed plane. Additional data on peripheral leaks can be gathered with a second laser sheet illuminating the transverse plane simultaneously^75^ or sequentially,^65^ or with a cone‐shaped illumination area^66^ illuminating the volume around the head of a manikin. The absence of visible particles in front of an investigated mask can result either from a low quality of fit, redirecting a large fraction of the particles upward with the leaking flow, or from a high filtration efficiency blocking the particles carried by the flow through the mask, or both. Complementary imaging via the Schlieren technique can provide supplemental information on the direction of the airflows escaping the mask for a better estimation of the fit and the filtration efficiency.

### 4.3 Thermal imaging

A thermal camera measures the changes in the temperature of the skin at the interface with the mask caused by the inward flow (temperature decrease from colder air entering the mask) and outward flow (temperature increase from warmer air flowing out).^76^ The ability to detect inward leakage constitutes a significant advantage of this technique over the methods described earlier which are only able to detect outward leaks. The method has been used to validate results from CFD simulations predicting the positions of leaking spots with N95 masks.^77^ However, thermal cameras have a limited resolution in leakage detection and cannot replace fit testing. They are limited to the detection of massive leaks.^78^ Leakage detection using infrared imaging can easily be applied to human volunteers as it does not require the use of tracers or smoke. However, the fast cooling of the exhaled flow limits the visualization to the immediate vicinity of the wearer's head. A more recent work combined a deep learning model with infrared thermography^79^ for a faster and automated detection of leaking airflows by comparing the temperature information with and without masks. Such a method would allow a more precise detection of the leaking flow compared with the unassisted estimation of the leaking flow from thermal mapping; but remains sensitive to ambient temperature and humidity.

### 4.4 Particles tracing

Fluorescent tracers can reveal the trajectories of particles and their deposition patterns on masks at inhalation^80^, ^81^ and exhalation.^31^ Fluorescent particles sprayed on surgical masks have been used to investigate the deposition patterns and estimate surface contamination.^82^ Fluorescent particles have also been used to assess the impact of gender, mask brand and exercise (movements of the body and the head) on the location and shape of faceseal gaps.^83^

### 4.5 Measurement of airborne particles concentrations

Direction and magnitude of leaking flows can be inferred from the measurement of the particles' concentration around the source, and to compare masks and protection devices^47^, ^84^ in their ability to contain the forward flow and limit leakage. Source control with surgical masks was found to reduce the release of particles by 70% for speaking and 90% for coughing.^85^ The measurements of the concentration around the emitter do not provide detailed information about the airflow or a precise identification of the leaking area at the mask/face interface but permit the identification of the areas where the emitted particles accumulate.

### 4.6 Synthesis of the most common leaking spots

Facemasks reduce the horizontal spread of the exhaled airflow and droplets, with the exception of respiratory protective devices equipped with an exhalation valve. The valve lowers the pressure drop at exhalation which reduces the peripheral leakage, directs all the flow forward, and releases it almost unfiltered,^62^, ^64^, ^75^ making such devices unsuited for source control.

Respiratory protective devices show lower levels of peripheral leakage than medical and community facemasks^58^, ^65^, ^66^ and divert a higher fraction of the flow through the filtering part before being released in front of the wearer,^58^, ^61^, ^64^ while medical and community masks direct a significant fraction of the exhaled flow toward the gaps, with the resulting unfiltered flow being released in the immediate vicinity of the wearer. However, respiratory protective devices do not fully contain the flow generated by violent expiratory activities such as coughing and sneezing.^58^, ^65^, ^72^ The forward leakage from respiratory protective devices depends on the fit of the mask,^84^ and peripheral leakage from these devices is mostly limited to the area around the nose and the cheeks.^65^, ^66^, ^72^, ^76^, ^77^

Various leaking spots have been reported on medical masks: downward around the chin, and sideward and backward near the cheeks,^47^, ^58^, ^60^, ^65^ that being said, the most common source of leakages is the nose area.^60^ A comparison between a nanofiber‐based surgical mask and a conventional surgical mask,^63^ as well as an investigation on different high‐efficiency inserts to improve the filtration efficiency of community masks,^86^ have shown that the pressure drop significantly influenced the level of leakage through the balance between the leaking flow and the flow directed to the filtering part.

The category community masks groups various types of filtering materials and designs (in which we also include homemade masks not necessarily following the guidelines for community masks) with a high variability in filtration efficiencies. The leaking behavior of different tested community masks is similar to medical masks with comparable leaking spots.^60^ Face shields do not filter droplets and only partially stop their forward motion. Most of the flow escapes the shields from the lower edge and has been shown to further spread forward after leaving the volume enclosed by the shield.^60^, ^72^, ^75^ Massive leakage was also observed in all the other directions, with significant concentration of particles measured at the back of the emitter.^47^, ^60^, ^72^, ^75^

A summary of the leaking spots is given in Figure 2 based on the nomenclature introduced by,^60^ with a typical leaking pattern for FFP2/N95 masks given in Figure 2C and medical/community masks given in Figure 2D. The positions of the leaking spots were compiled from^60^, ^62^ from light scattering and Schlieren optical technique. The FFP2/N95 masks showed a larger flow directed to the front (through the filtering part of the mask) in the Schlieren imaging,^58^ while laser visualizations reported a lower scattering intensity^73^, ^75^ indicating that the forward flow contains significantly less particles than the leaking flows as a result of filtration.

## 5 MODELING INWARD AND OUTWARD LEAKING FLOWS

### 5.1 Computational fluid dynamics

Computational fluid dynamics (CFD) is a powerful tool to model the spread of the airflow and the carried droplets through and around different types of facemasks. While the techniques to visualize the airflow provide valuable information, modeling supports further investigations on the impact of different parameters (exhalation pattern, facial features, particles size, etc.) and of mask designs at a reasonable cost. CFD has been used to quantify the partition of the inhaled airflow between the flow going through the filter media and the flow through the leaking spots,^87^ with a focus on the impact of the gap area considering various positions of leaking spots along the faceseal interface. The model showed that a gap of 1 cm^2^ was sufficient to drive 17% of the flow through the leaks, thus being unfiltered (a gap of 4.3 cm^2^ led to 60% of the inhaled flow going through the leaks). The model only quantified the airflow and not the fraction of particles able to follow the leaking flow, as particles' ability to follow the leaking flow with strong curvatures depends on their diameter via the relaxation time. While small particles easily follow the flow, large particles might stick to the mask instead due to their hefty inertia. The transition between both behaviors depends on the flow velocity.

A focus on the particles going through the mask or escaping the leaking flow has been further investigated with the help of a Eulerian–Lagrangian multiphase model considering particles evaporation, breakup, and turbulent dispersion, to simulate the protection mechanisms of masks used for source control and respiratory protection.^88^ The leaks have been approximated with a fixed gap between the mask and the wearer's head creating leaking flows at the cheeks, the chin, and around the nose. The horizontal velocity of the exhaled airflow and the spread of the particles through the mask were significantly reduced compared with the case in absence of mask and particles were redirected toward the leaking flows. A similar representation of the gap (uniform gap of 2 mm along the faceseal interface) has been used to investigate the trajectories of exhaled particles in a conference room and to highlight the role of ventilation in the dispersion of the particles.^89^ Such models would benefit from a more realistic representation of the gaps along the interface between the mask and the wearer's face, as we highlighted in the previous sections that the leaking spots are not homogeneously distributed and that the area around the nose is more prone to leakages. Consideration of non‐uniform gaps could provide a more accurate description of the leakages. For example, a CFD simulation to describe the spread of contaminated droplets in a ventilated room considered gaps between 1 mm on the sides and 6 mm around the nose.^90^ The simulation has shown a drop of the mean diameter of the released particles with mask, as large particles were well filtered by the filter media and tended to stick to the mask instead of following the leaking flow. The fate of the leaking particles will be discussed in the following paragraphs with the help of various models and simulations.

The protection provided by neck gaiters, cloth masks, and face shields and the corresponding leaking flows have been modeled as a function of the particles' diameters,^91^ differentiating particles collected by each protective device from particles escaping through the leaks. The leaking spots have been calculated from the interaction between the 3D models of the wearer's face and the corresponding mask. The neck gaiter showed the best ability to limit the leakages as it can be tightly wrapped around the face but had the lowest filtration efficiency. The cloth mask had a significantly higher leaking flow than the neck gaiter, which was countered by a higher filtration efficiency. The face shield showed massive outward leakages, significantly larger than both the neck gaiter and the cloth mask, and efficiently stopped only larger particles (d > 30 μm) while smaller ones followed the leaking airflow and escaped unfiltered. All the protections have shown a similar cutoff size of particles able to follow the outward leaking flow, with smaller particles (diameters smaller than 10–30 μm) easily following the escaping flow and larger particles (>30–40 μm) being caught on the filtering material.

Another CFD simulation focused on the fate of particles (stick, penetrate, or follow the leaking flow) interacting with surgical and cloth masks,^92^ showing that a high fraction of smaller particles (d < 20 μm) were able to follow the leaking flow, while larger particles rather stuck to the mask as they could not follow the curvature of the leaking flow. Results were similar to the previously mentioned model^91^: less particles were able to leak from cloth masks, which was compensated by a higher penetration through the filter media.

### 5.2 Analytical and numerical models

Analytical and numerical models have been applied to calculate the leaking fraction as a function of various parameters, such as the resistance generated by facemasks against the inhaled or exhaled airflows, or the cross‐section of the leaking spots. The leaking fraction as a function of the total flow penetrating a mask at inhalation and exhalation has been derived from the pressure drop generated by both the mask (determined by the resistance of the fabric) and the gaps (determined by its dimensions) in an analytical model.^93^ The framework has been validated with complementary CFD modeling providing additional data such as the pressure and velocity distribution within the volume enclosed by the mask and the wearer's face. A similar work proposed an analytical method combined with CFD modeling to derive the filtration ratio and the fit factor as a function of the pressure drop and the cross‐section of the gap.^94^ The relation between pressure drop and leaking flow has been applied in an estimation of the flow going through the leakages of various types of masks (surgical and community masks) as a function of the measured pressure drop.^95^ The measurements of the pressure drop have been performed on a dummy head and showed that a significant fraction of the exhaled flow escaped from the gaps. These numerical methods have been initially developed to estimate the leaking airflow without considering the particles. The methods can be incorporated into models focusing on the particles carried by the leaking flow.

The total inward leakage as a function of the diameter of the penetrating particles has been investigated with an analytical model complemented by CFD.^96^ A N95 mask has been approximated by a spherical porous layer and the faceseal leakage by an annular peripheral opening between the mask and the wearer's face. Particles between 10 nm and 1 μm have been considered in the calculations, leaving larger particles out of the scope of the study. Even small gaps have been found to significantly degrade the total protection efficiency: a gap representing 0.1% of the mask's surface was sufficient for a N95 respirator to create an inward leakage larger than 5% from leakage alone (penetration through the filtering material not considered). A leak area >1.5% of the total mask/face contact area would drive the total inward leakage of a N95/FFP2 mask above 20%.^94^

These models did not take into account additional parameters such as the surface roughness of the skin or the mask, nor did they consider more complex geometries of the leaking spots. The resulting leaking fraction might therefore be overestimated, as a more tortuous and rougher leaking path might increase the pressure drop, and thus, modify the distribution between the mask flow and the leaking flow. The roughness of the wearer's skin, together with the contact pressure of the mask along the contact surface and the elasticity of both the mask and the skin have been considered^97^ under the assumption of a uniformly distributed contact pressure, to calculate the resulting gaps between the skin and the mask. This assumption was appropriate only with masks allowing a tight fit on the wearer's face, such as a N99/N95 and FFP2/FFP3 masks.

### 5.3 The role of facial features in the faceseal leakage

The position and size of the gaps are significantly influenced by the size of both the mask and the face, as well as numerous facial features, such as the size of the nose, the dimensions of the cheeks, or the distribution of soft (muscles and fat) and hard tissues (bones). Movements of the head, movements of the facial muscles to smile and to express sadness, anger, or surprise dynamically change the contact at the interface between the face and the mask, thus influencing the fit factor. An algorithm has been developed to compute the contact area between N95 and headforms with different sizes.^98^ Models simulating the donning of a mask have been created to derive the impact of face morphologies and mask parameters (size, pleating, etc.) on the contact area and the gaps,^99^, ^100^ highlighting the benefits in protection efficiency from adapting masks to different types of faces. Computed tomography (CT) has also been deployed on headforms equipped with N95 masks to visualize the contact area and the gaps,^101^ evaluating in total nine combinations of different mask and head sizes. The resulting 3D data have been integrated into a CFD model to calculate the relation between the inward leakages and the gap's surface area. The SARS‐CoV‐2 infection risk has been calculated using a lung deposition model and an SIR (Susceptible, Infected, Recovered) epidemiological model. The mask/headforms pairs have been found to reduce the infection risk from 97% (no mask) to 42%–80% with respirators and up to 12% for fit‐tested respirators (assuming a leaking fraction equal to the maximum allowed by the corresponding standard). A virtual headform has been reconstructed from the CT scan of a volunteer's head to feature realistic distributions of soft and hard tissues leading to a precise calculation of the deformations.^102^ The headform has been integrated into a mask‐wearing model to analyze contact areas and formation of gaps with a N95 respirator, as well as to derive the contact pressure as an indicator for mask wearing comfort. The model has led to the identification of area prone to leakage, confirming previous findings (the nose region, the cheeks, and the chin). The method has been further used to calculate the deformation induced by facial expressions and the corresponding changes in contact pressure.^103^ The impact of head movements (moving up and down, rotating) has been modeled with a dynamic headform^42^ based on observation of real movements. The subsequent contact simulation^43^ has led to the calculation of the evolution of the contact pressure at different points of the mask/head contact area upon head movements.

The importance of adapting masks to facial features has been experimentally highlighted through the quantification of the additional protection provided by a 3D‐printed frame, developed to provide a better fit of the mask on the wearer's head.^104^ Without modifying the filtration properties of the filter media (taken from surgical masks), the frame increased the fit factor from an average value of 4.4 to an average of 158 on five wearers performing head movements similar to those required in the fit test as described by OSHA, bringing the level of protection close to those required from N95 respirators. However, significant differences have still been measured between masks as well as a high variability between the different users.

### 5.4 Role of the leakage in physical models describing the spread of exhaled droplets

Numerous computational models have been developed to simulate the impact of mitigation measures (wearing masks, social distancing) on the spread of SARS‐CoV‐2. Combining the emission distributions of various expiratory activities, the trajectories of emitted particles, their evaporation and interaction with local turbulences, their filtration by the emitter's and the receiver's masks, and their subsequent deposition into the respiratory tract, Schmitt and Wang^105^ have calculated the protection provided by facemasks under various leaking scenarios to highlight the importance of the leakage in the estimation of the overall protection efficiency and have quantified the exposure to infectious respiratory droplets considering the pressure drop of various types of masks. The infection probability has also been derived as a function of the size of gaps between masks and the wearer's head.^106^ Both models^105^, ^106^ based their estimation of the leaking flow on an analytical model mentioned earlier.^93^ A numerical model describing the size‐dependent collection efficiency of an impactor has been integrated to estimate the fraction of particles not able to follow the leaking airflow^105^: a cutoff size has been calculated, corresponding to the upper size limit for particles able to follow the leaking airflow; larger particles would collide on the filter. This cutoff size was dependent on the flow velocity (which was dependent on the level of leakage) and showed a good agreement with previously mentioned investigations based on CFD^91^, ^92^ with cutoff sizes around 20–30 μm.

A model estimating the upper limit of the infection risk faced by an individual interacting with an infected person has been developed, based on measurements of the size‐dependent total inward particles penetration on volunteers wearing various types of masks under different fitting conditions.^107^ The role of facemasks used as respiratory protection in the reduction of the infection risk^108^ has been found to be dependent on the concentration of viruses in the vicinity of the mask‐wearing individual (distinguishing virus‐rich and virus‐limited environment), as a result of the non‐linearity of the dose–response relationship. The calculations considered the total inward penetration of N95 and surgical masks obtained from data available in the literature. A physical model based on a multidisciplinary approach to derive the infection risk considered a uniform outward leakage (15% for both surgical masks and respiratory protective devices) combined with realistic size‐dependent filtration curves gathered from the literature.^109^ The total inward penetration has been derived from the standards for respiratory protective devices and set to a uniform distribution between 83% and 91%. The inward penetration for surgical masks was based on the available literature and set to a uniform distribution between 25% and 80%. In an indoor scenario simulator developed to investigate the impact of several parameters (room size, ventilation rate, type of mask, exhaling activity) on the infection risk,^110^ FFP masks have been assumed to pass the fit test and the inward leakage has therefore been taken from the EN 149 standard. An overall protection efficiency of 80% has been applied to all masks at exhalation, with the exception of masks with a valve set to an outward protection of 5%. Neither the inward nor the outward protection has been assumed to be dependent on the particle diameter. A similar quantification of the leaks^111^ assumed a protection efficiency of 30% for masks used as respiratory protection, and 60% in source control. A high‐efficiency mask (95% filtration) has also been included. In an estimation of the mask efficiency to reduce the horizontal spread of droplets, Wang et al.^112^ have represented the leaking flow as a fraction of the particles not being filtered by the mask, which has been based on experimental values available in the literature.

Other models^113^, ^114^ have only dealt with the filtration efficiency of the mask, assuming a perfect fit on the wearer's face. As the leakages account for the largest fraction of particles entering (respiratory protection) or released (source control) via the mask, such a hypothesis is likely to lead to an over‐estimation of their protection.

### 5.5 Integration into compartmental epidemiological models

Compartmental epidemiological models dynamically assign a given population into different compartments depending on their epidemiological status. They do not focus on the physical phenomena occurring between an emitter and a receiver (droplets transport, particles emission, etc.) but rather test different hypothesis affecting the infection risk to predict their impact on the spread of the disease. An SIR model (Susceptible, Infected, Recovered), with the acronym corresponding to the different compartments the individuals can be assigned to, has been developed to investigate the impact of the protection provided by facemasks (leakage and filtration efficiency were merged into a protection coefficient, similar for inhalation and exhalation) and the fraction of the population wearing masks on the effective reproduction number.^115^ A SEIR model (adding the Exposed compartment) has been proposed to estimate the hospitalizations peak and the mortality as a function of the masks' efficacy (including filtration and leakage) and usage.^116^ A SEAIR model has additionally considered asymptomatic individuals^117^ to compare the impact of mask wearing and social distancing on the mortality and the incidence. The masks have been modeled through a reduction of the probability of transmission by 95% if the mask was worn by the infectious emitter, and by 85% if it was worn by the receiver.

## 6 ESTIMATION OF THE IMPACT OF MASK LEAKAGE ON THE INFECTION RISK WITH CONSIDERATION OF VACCINATION AND VIRUS VARIANTS

The synthesized information on mask leakage was applied to evaluate the infection risk in different scenarios including the influence of vaccination and variants of SARS‐CoV‐2. Three types of masks were modeled: a FFP2 mask, a medical mask, and a community mask each with realistic levels of leakage taken from the compiled data presented in Figure 1. The filtration efficiency curves of the masks are given in Appendix S1. Three levels of leakage (low, intermediate, and high) were considered: the low leakage level corresponded to the 5th percentile of the values given in Figure 1 (i.e., 5% of the population was expected to have a fit factor lower than this value), the high level to the 95th percentile, and the intermediate level to the median value. The resulting leaking fractions for each mask are indicated in Table 2. The leakage was given as the fraction of the total exhaled (mask on the emitter) or inhaled (mask on the receiver) air stream that flows through the gaps between the mask and the wearer's head. We considered that the leaking flow escaped around the nose in an upward vertical direction (crown leak according to the nomenclature presented in Figure 2) as this configuration posed a higher risk than back or side leaks, assuming the receiver in front of the emitter. The three scenarios feature the same numerical values for the inward and the outward leakage. The outward leakage is applied to the emitter and the inward leakage is applied to the susceptible receiver.

**TABLE 2:** Levels of leakage adopted for the three types of masks

	FFP	Medical	Community
Low level	0.39	0.54	0.9
Intermediate level	0.03	0.24	0.42
High level	0.005	0.09	0.093

*Note*: The values indicate the flow through the leaking spots as a fraction of the total flow. The same values were adopted for inward and outward leaks. The low level corresponded to the 5th percentile of the fit factors presented in Figure 1, the intermediate level corresponded to the median value, and the high level corresponded to the 95th percentile.

Abbreviation: FFP, filtering face pieces.

The infection risk was calculated in three scenarios describing realistic interactions between an infected emitter and a receiver. The *Indoor* setting was considered to be the reference as both individuals were interacting (speaking and breathing) for 15 min and separated by 1 m, which corresponded to the guidelines for contact tracing given by the World Health Organization.^118^ No ventilation was considered in this setting. The *Office* scenario simulated both individuals breathing and speaking 5 m apart from each other for 8 hours considering a ventilation rate of 5 air changes per hour (guidelines recommended 0.35–8 air changes per hour for indoor spaces^119^). The *Hospital* scenario simulated the interaction between an infected individual and a healthcare worker, assuming a 1‐hour interaction at 1 m and including breathing and coughing (1 cough per minute), with an increased ventilation rate (10 air changes per hour). The infection risk was calculated with a computational framework we developed in a precedent work,^105^ taking into consideration the variability in the total number of emitted droplets, the concentration of viral charges in the liquid fraction of the droplets, and the uncertainties over the dose–response relationship. Details are available in Appendix S1.

**FIGURE 3:**
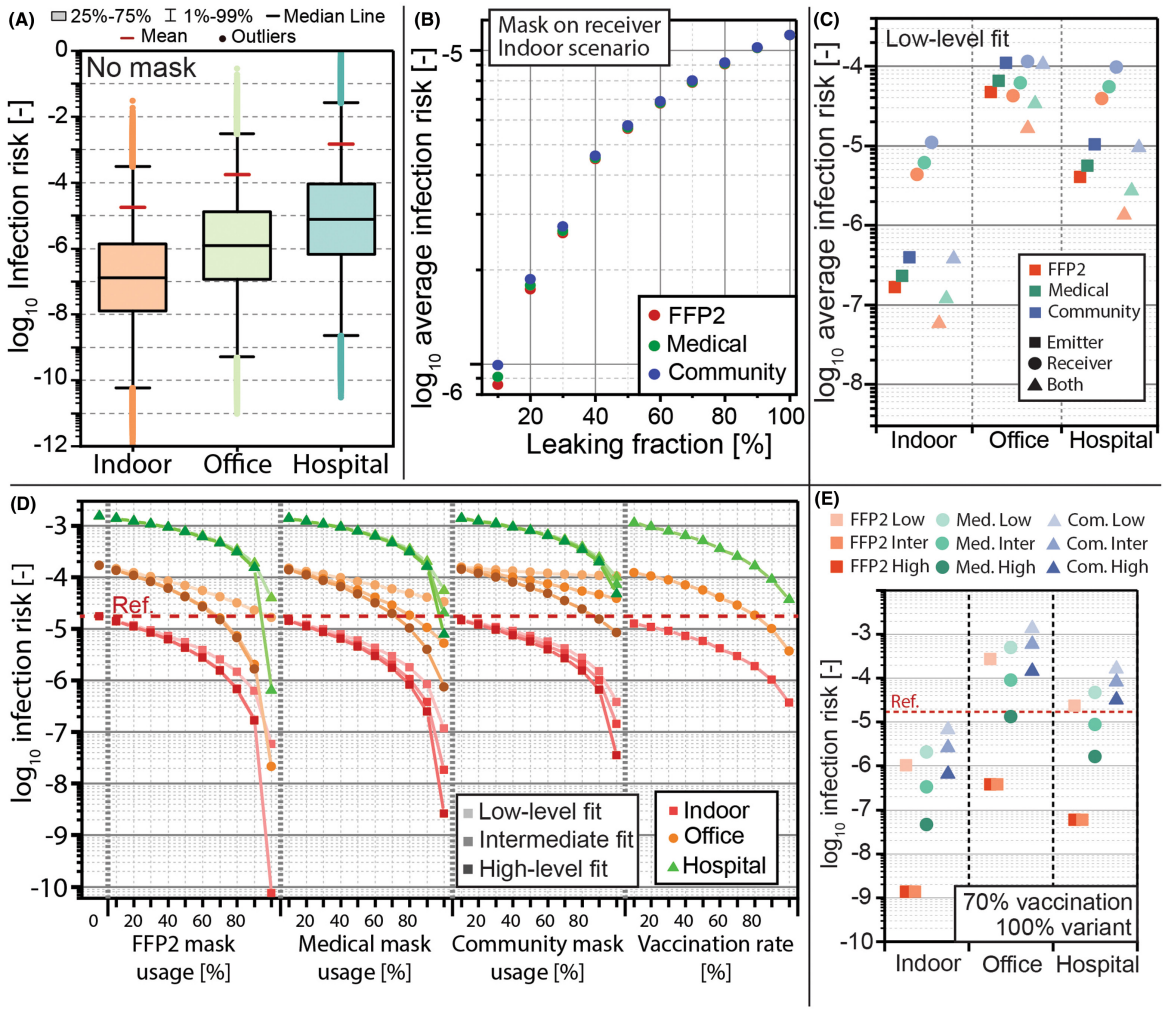
Potential of masks to mitigate the infection risk in various situations. The risk distribution without a mask is given in (A); the average risk as a function of the leaking fraction for FFP2, medical, and community masks worn by the receiver in the indoor scenario is given in (B); the average infection risk in the different scenarios with masks on the emitter, on the receiver, or on both is given in (C) considering the low‐level fit scenario; the adoption of facemasks with the three realistic levels of leakage described in Table 2 was compared with the vaccination rate and shown in (D). The calculations considered a random encounter with an infected emitter and a receiver, each one having a probability equal to the mask usage rate to wear a mask, or equal to the vaccination rate to be vaccinated. The reference for the calculation of the relative infection risk was the average risk in the *Indoor* scenario without mitigation measures. The combined mitigation impact of vaccination (70% vaccination rate) and masks, with the assumption that 100% of the infected individuals carried the variant (Delta variant, additional data in Appendix S1) and that both individuals wore masks, is given in (E). The infection risks for the three masks (FFP2, medical—Med., and community—Com.) considered for the three levels of fit (low, intermediate, and high).

The distribution of the infection risk in the absence of mitigation measures is given in Figure 3A. The *Hospital* scenario led to the highest infection risk with a significant contribution from the coughs of the infected patient (accounting for 91% of the infection risk). The *Indoor* scenario led to the lowest infection risk. The *Office* scenario generated a higher infection risk than the *Indoor* scenario as a result of the longer interaction time despite the larger distance. All three scenarios showed a wide distribution of the infection risk spreading over 10 orders of magnitude as a result of the distributions of input parameters. We therefore based the calculations of the impact of mitigation measures and variants on the average values. The variation of the infection risk in the *Indoor* scenario as a function of the leaking fraction for FFP2, medical, and community masks worn by the receiver is given in Figure 3B. It is worth noting that while for a low level of leakage (<30%) the type of mask made a noticeable difference in the infection risk, for higher levels of leakage the differences in mask filtration efficiencies were attenuated because the leaking flow dominated, and the masks could only be differentiated by the quality of the fit they were able to provide. The influence of the three types of masks (FFP2, medical mask, and community mask) on the infection risk considering the low level of fit as given in Table 2 is shown in Figure 3C for masks used as source control, respiratory protection, and both. Source control had advantages over respiratory protection as it slowed down the particles and the airflow penetrating the mask and diverted the leaking flow away from the receiver. Therefore, most scenarios demonstrated a significantly lower infection risks for source control over respiratory protection. However, the *Office* scenario constituted one exception with a slightly lower infection risk through respiratory protection compared with source control (−10% for a FFP2 in the low‐fit scenario). A detailed discussion is available in Appendix S1.

The variation of the relative infection risk as a function of the usage of FFP2, medical and community masks with the three leakage scenarios, and as a function of the vaccination rate (without a mask) is given in Figure 3D. The mask usage indicated the fraction of the population wearing masks and determined the probability that the emitter, the receiver, or both wore a mask in the given scenario. In a similar way, the vaccination rate indicated the probability that the emitter, the receiver, or both were vaccinated. The effect of vaccine was modeled with lower infectivity and viral charge concentration (details in Appendix S1). The mask usage in the *Hospital* scenario was applied only to the receiver, as the emitter was considered to be a hospitalized patient under treatment and did not wear a mask. Mask usage significantly reduced the infection risk in all scenarios, but a high FFP2 and medical mask usage rate combined with a high level of fit were required to bring down the risk in the *Office* and *Hospital* scenarios below the reference value (the differences between the intermediate and the high‐level fit for the FFP2 mask were not noticeable on the graph as the corresponding leaking fractions were close). A community mask, even with a high level of fit, was not sufficient to bring the infection risk in the *Hospital* scenario down to the reference level. In the absence of a mask, a vaccination rate >80% would have been required in the *Office* scenario to reach the reference level, but it would not have been reached in the *Hospital* scenario even with a 100% vaccination rate. A combination of vaccination and mask usage would therefore have been necessary. The FFP2 mask was able to significantly reduce the infection risk with a high level of fit compared with a low level (3 orders of magnitude at 100% mask usage), while the differences were reduced for medical masks (about 2 orders of magnitude) and community masks (1 order of magnitude).

The combined mitigation impact of vaccination and masks is given in Figure 3E, with the assumption that 100% of the infected individuals carried the Delta variant (which was the case in most of the countries tracking variants in new infections before the emergence of the Omicron variant at the end of 2021^120^), that both the emitter and the receiver wore the masks, and 70% of the population was vaccinated. The variant was modeled with an increase of the infectivity, an increase of the concentration of viral charges, and a reduction of the efficiency of vaccination (details in Appendix S1). As defined earlier, the reference infection risk corresponded to the average risk in the *Indoor* scenario without a mask, with the original virus strain, and without vaccination. A high level of fit was required to bring the infection risk down to the reference value, emphasizing the importance to combine vaccination and mask usage. Masks were sufficient to compensate the impact of the variant in the *Indoor* scenario, whereas at least an intermediate‐level fit with a FFP2 or with a medical mask was required in the *Hospital* scenario, while the intermediate‐fit on a medical mask was not sufficient in the *Office* scenario. The targeted reduction of the infection risk could not be achieved with a community mask in the *Office* and *Hospital* scenarios.

The need for a high adoption rate of masks possessing a high level of fit was found to be even more critical with the latest Omicron variant. Early reports suggested a significantly higher viral charge in the upper respiratory system (10 to 100‐fold increase) than the Delta variant,^121^ a higher infectivity,^121^ and a reduced efficiency of vaccines.^122^, ^123^ Calculations in the *Indoor* scenario without mitigation measures indicated a 3.4 × 10^3^ times higher infection risk compared with the original strain and 40 times compared with Delta. Mask wearing (intermediate‐fit medical mask) and vaccination (70% vaccination rate) could reduce the risk to 6 times the reference risk and a vaccine booster shot could bring the risk down to 2.8 times the reference risk. Details can be found in Appendix S1.

## 7 LIMITATIONS OF THE DEVELOPED MODEL

The model presented in this work was used to compare the impact of mitigation measures (masks and vaccination) on the infection risk in different scenarios, as well as to consider variants with an increased infectivity. However, the model had several limitations. The size distribution of the emitted droplets had a significant impact on the infection risk and on the evaluation of the protection provided by facemasks, as shown by a comparable model considering a slightly different emission size distribution.^124^ As the amount of viral charges in a particle was considered to be mostly dependent on the particle's volume, large particles can have a significant impact on the calculation of the infection risk and on the quantification of the mitigation role of facemasks. However, such particles are challenging to measure as they have substantial settling velocities, making them less likely to reach the sampling instruments. The calculation of the infection risk is based on the dose–response relationship and on the concentration of viral charges in the emitted droplets. Both parameters are still under investigation for SARS‐CoV‐2, with recent publications focusing on quantifying the viral load in the exhaled breath.^125^, ^126^, ^127^ Our estimation for the infectivity is based on the SARS‐CoV‐2, as detailed in Appendix S1. Changes to these parameters can significantly impact the infection risk. The impact of vaccination and variants was simplified, and only average values were considered. Research on this topic is still ongoing and new data are regularly published. The impact of vaccination has been shown in the literature to be strongly dependent on the age of the involved individuals, as well as the time since their vaccination, and the type of vaccine injected, which were not considered in this model. The calculated infection risk did not differentiate an asymptomatic infection from an infection leading to mild symptoms or to a hospitalization. We aimed at taking into account the variability of the input parameters such as the total number of emitted droplets, the viral charge concentration and the infectivity (still under investigation for SARS‐CoV‐2 so far and highly dependent on new variants) and calculated infection risk distributions spanning over several orders of magnitude. We also based our calculations on estimated fit factors for the masks, intended to reflect the fitting qualities of FFP2 and medical masks worn by non‐trained users. However, as we highlighted in the review part, the fit factor is likely to show a high variability and is influenced by numerous parameters such as facial features or dynamic phenomena like coughing or sneezing. Finally, we considered in our scenarios that the inward leakage was equal to the outward leakage, as most of the data available in the literature to quantify the level of leakage focuses on inward leakage. This may lead to inaccuracy, as the outward leakage is likely to be higher than the inward leakage due to the higher pressure of the air enclosed between the mask and the wearer's face at exhalation, which might create additional leaking spots comparing to the lower pressure scenario at inhalation.

## 8 CONCLUSION

In the present work, we reviewed various aspects of facemask leakage and synthesized them to calculate the mitigation effect of masks with realistic levels of leakage. The different standards only partially regulate the level of leakage, as masks are used for both respiratory protection and source control, and therefore, outside the scope of usage they were designed for. Measurements of the protection factor in realistic conditions have demonstrated a high variability, due to the skills of the wearers in properly adjusting their masks, potential mismatches between mask and face sizes, and movements of the head. Various methods have been developed to identify the areas prone to leakage and we summarized typical leaking patterns for filtering facepieces, medical, and community masks.

Simulation complements measurements as it provides a tool to investigate the impact of various parameters (i.e., facial features, movements of the head, breathing resistance of the mask) on the protection provided by a mask. Including the leakage in physical transmission and compartmental epidemiological models is crucial to provide a realistic estimation of the protection provided by masks and their impact on the spread of the disease.

The information gathered on leakage was summarized and integrated into a computational model to evaluate the ability of masks in reducing the infection risk assuming realistic levels of leakage and to compare the protection provided by masks with vaccination taking new SARS‐CoV‐2 variants into account. The results indicate that a high adoption of facemasks and a proper fitting are required together with vaccination to limit the infection risk, especially in the face of the emerging SARS‐CoV‐2 variants. The differences in protection efficiencies between the three levels of leakage presented in the last section of this work and likely to be found within a population of non‐trained users highlight the significant gains in protection that could be obtained with improvements of the fit on the wearer's face, for example, with the help of a flexible frame that would better adapt the filter media to the facial features. The protection efficiency would benefit from a focus on improving the fit rather than the filtration properties of filter media.

The model presented in this work can be adapted to potential future diseases transmissible through respiratory droplets, and the information compiled in the review section can benefit the development of masks more suitable for a general use within a population of non‐trained users to slow the spread of future pandemics.

## AUTHOR CONTRIBUTIONS

J.W. conceived the study. J.S. reviewed the literature, gathered the data, and developed the model's code. J.W. and J.S. wrote the paper. All authors approved the final version of the manuscript.

## CONFLICT OF INTEREST

The authors report there are no competing interests to declare.

## Supporting information


Appendix S1

## Data Availability

The data that support the findings of this study are available from the corresponding author upon reasonable request.
