# Supplementary material for: A critical review on the role of leakages in the facemask protection against SARS‐CoV‐2 infection with consideration of vaccination and virus variants
Source: Indoor Air. 2022 Oct 11;32(10):e13127. doi: 10.1111/ina.13127 (PMC9828278; doi:10.1111/ina.13127)
Supplement: Supplementary file 1 — Appendix S1 [file INA-32-0-s001.docx]

**A critical review on the role of leakages in the facemask protection against SARS-CoV-2 infection with consideration of vaccination and virus variants**

**Supporting Information**

Jean Schmitt^1,2^ and Jing Wang^1,2*^

^1^Institute of Environmental Engineering, Department of Civil, Environmental and Geomatic Engineering, ETH Zurich, Zurich 8093 Switzerland

^2^Laboratory for Advanced Analytical Technologies, Empa, Swiss Federal Laboratories for Materials Science and Technology, Dubendorf 8600, Switzerland

*Correspondence: [jing.wang@ifu.baug.ethz.ch](mailto:jing.wang@ifu.baug.ethz.ch)

**Table of contents**

1. Discussion on the impact of a reduced sampling volume
2. Filtration efficiency of the modeled facemasks
3. Variability of the initial parameters and modification to the computational model
4. Source control versus respiratory protection in the *Office* scenario
5. Modeling the impact of vaccination and variants
6. **Discussion on the impact of a reduced sampling volume**

Measurement of the protection efficiency versus source control for masks worn by manikins in a closed volume performed by Pan et al. [1] highlighted that source control did not provide a significant advantage over respiratory protection. Other measurements [2] reported a significant advantage of source control over respiratory protection. We attributed the differences between both conclusions to differences in the test volumes: Pan et al. [1] uses a volume of 51x34x33 cm^3^, Mansour and Smaldone [2] considered a volume of 158x150x189 cm^3^ with an integrated ventilation system providing 6 air changes per hour.

We implemented the similar parameters into our computational model (distance 33 cm, interaction time of 30s and room volume of 51x34x33 cm^3^) and compared the results to the *Outdoor 1* scenario which does not have any volume limitation. We found that the protection provided by source control was significantly lower with the parameters used by Pan et al. [1] compared to our *Outdoor 1*. Source control reduced the infection risk to 85% of the no-mask value while respiratory protection reduced it to 94% of the no-mask value, while it was significantly more efficient in the *Outdoor 1* scenario with a reduction to 3% of the no-mask risk compared to 20% for respiratory protection.

1. **Filtration efficiency of the modeled facemasks**

The filtration efficiency curves of the facemasks considered in the modeling section were reconstructed from measurement available in the literature. The reconstruction algorithm matched the experimental datapoints with the theoretical model describing the filtration efficiency of facemasks. Details on the algorithm are given in Schmitt and Wang [3]. Experimental data for the reconstruction of the masks are taken from the EN 149 standard for the FFP2 mask, from Oberg and Brosseau [4] and Drewnick et al. [5] for the surgical mask, and from the CWA 17550 for the community mask. The corresponding filtration curves are given in **Figure S1**.


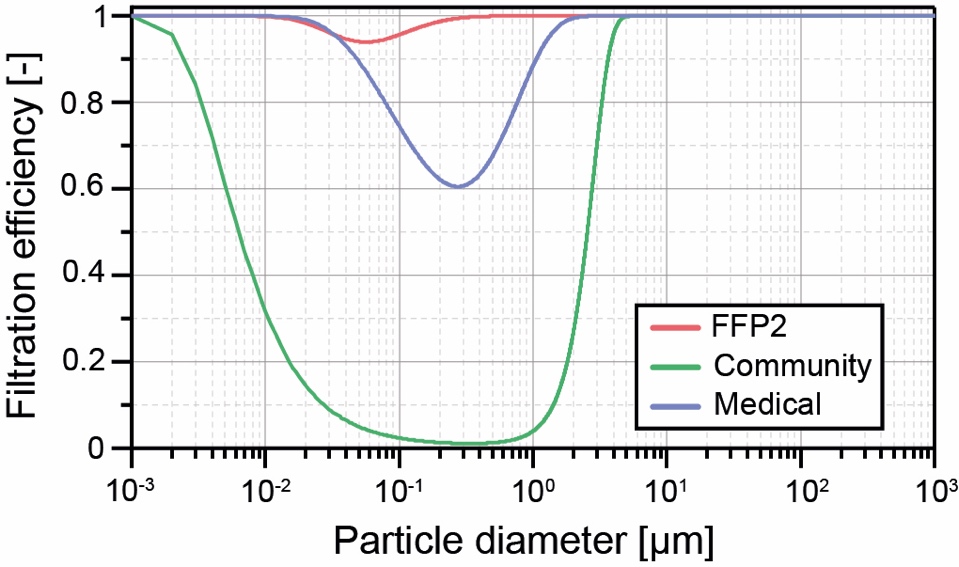


**Figure S1**: Filtration efficiency curves of the three face protections considered in the calculation of the risk of SARS-CoV-2 infection.

1. **Variability of the initial parameters and modification to the computational model**

The computational model was based on the framework developed by Schmitt and Wang [3], modified to feature a dose-response relation to calculate the infection risk, and realistically reflect the variability in the total number of emitted droplets for each expiratory activity, the variability in the concentration of viral charge among infected individuals, and the uncertainty over the virus’s infectivity.

C.1. Dose-response relationship

The dose-response relationship link the inhaled viral charge and the infection risk. The infection risk was calculated according to Equation 1. $p$ is the infection risk, $C_{v}$ the viral charge and $k$ the dose-response relationship.

$$\begin{aligned} p=1-e^{- \frac{C_{v}}{k}}\#1 \end{aligned}$$

C.2. Modification of the calculation of the inhaled viral charge

The volume occupied by the emitted particles, also called “particles cloud”, was derived from their trajectories to estimate the concentration of viral charges in the volume of air inhaled by the receiver. The displacement and extension of the volume under the influence of the emitted plume and the ambient air movements was dynamically calculated during the time of the interaction. A summary is presented in **Figure S2**. The near-field and far-field viral exposure calculated in the original model were merged into the calculation of the particles clouds to provide a more realistic overview of the concentration of viral charges in the ambient air by removing the assumption of a homogeneous aerosol concentration, which is not valid for short interaction times.

During each time step, the positions of approximately 400 particles of each size (36 size bins considering 10 points per decade between 200 nm and 1 mm) were recorded and the minimal, average, and maximal positions were extracted to define the cloud of particles. One cloud was calculated for each size class and time step, and was defined by its volume and position (as shown in **Figure S2a**). The volume was derived from the positions by considering 8 partial ellipsoids, one covering each combination of half-axes. Once the velocity of the exhaled air dropped below a certain threshold (1 mm/s), the spread of particles clouds was not based on individual particles anymore but on the extension of the entire cloud considering diffusion, gravitational settling, and movements of the ambient air (as shown in **Figure S2b**). The viral charge concentration in each cloud was calculated from the particles present in the cloud and its volume. Single particles were be excluded from the cloud as they settled on the floor or fell below a certain altitude threshold. These particles were not considered in the calculation of the cloud’s volume and viral charge concentration anymore. This was necessary for particles around 100 μm that are still too small to follow a ballistic trajectory and leave the airflow along its trajectory and do not reach the receiver.


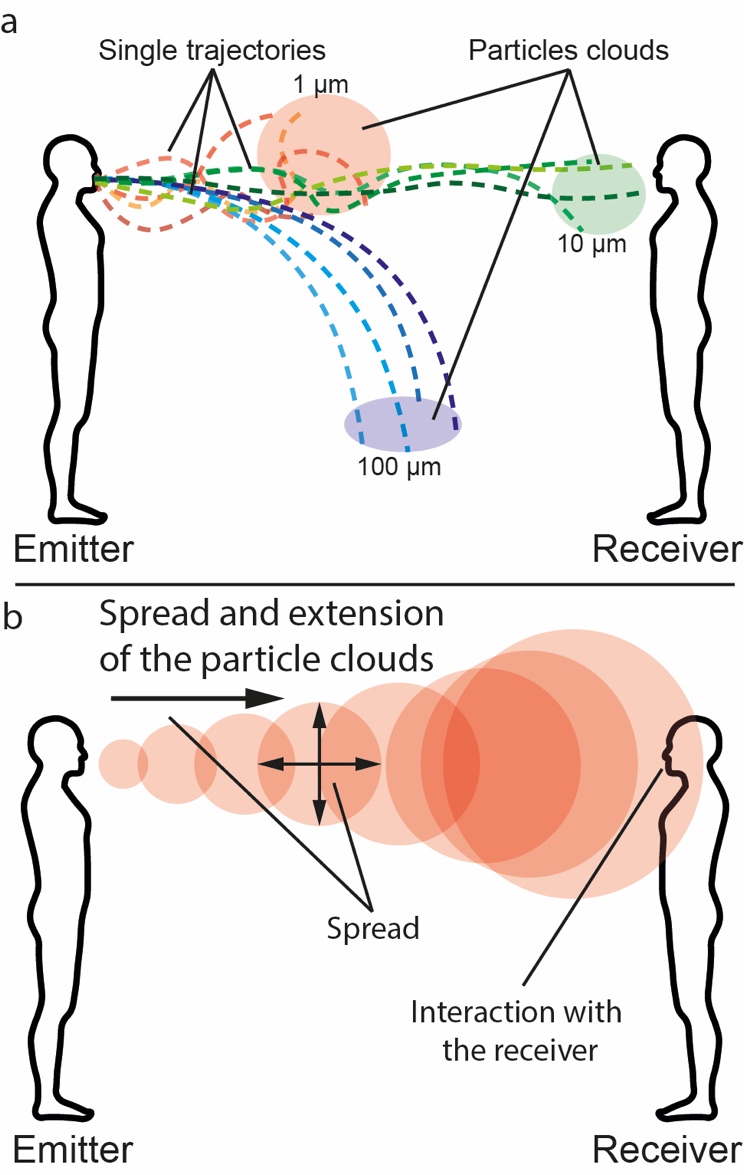


**Figure S2**: Illustration of the calculation of the viral charge concentration. The method for the calculation of the volumes of the particles clouds is given in (a), and their spread is given in (b).

C.3. Changes in the calculation of the centerline velocity

The emitted airflow was based on a turbulent jet model and described by a centerline velocity which was used to calculate the streamwise and radial velocities. The jet model originally used in Schmitt and Wang [3] was modified to take into account the transient emission velocities considered in the expiratory activities. Breathing and speaking were considered to generate a flow during 2 s before the initial velocity dropped to zero. A similar initial flow was applied for sneezing and coughing, but the emission time was reduced to 0.5 s. As a consequence, the emitted jet could not reach its steady state, which is a hypothesis of the original jet model. A more realistic calculation of the centerline velocity was derived from computational fluid dynamics (CFD) based on a Reynolds-averaged Navier-Stokes (RANS) k-ω model using the software COMSOL Multiphysics.

The centerline velocity calculated with the turbulent jet model for the four expiratory activities was compared to the CFD modeling in both steady-state and transient state considering an exponential decay of the emitted velocity to represent the dynamic of the emission activities. The CFD simulation was done using the 2D turbulent flow model in Comsol Multiphysics. The domain was represented by a rectangle with a height of 2 m and a length of 10 m. The source was represented by a 0.02 m line. The following initial and boundary conditions were defined:

- Domain initial state: 0 m/s air velocity and pressure set to 10^5^ Pa
- Source: outlet, time-dependent flow oriented in the positive x direction
- Boundaries: outlet, condition of fixed pressure at 10^5^ Pa (3 boundaries) and wall with no slip condition (1 boundary)

The turbulences were modeled using the Reynolds-averaged Navier-Stokes (RANS) k-ω model. The initial velocity was defined by a stepwise function with the following parameters:

- Breathing: 2s at 1.6 m/s before drop to 0 m/s
- Speaking: 2s at 3.9 m/s before drop to 0 m/s
- Coughing: 0.5s at 11.7 m/s before drop to 0 m/s
- Sneezing: 0.5s at 20 m/s before drop to 0 m/s

A visualization of the simulated airflow produced by a sneeze is given in **Figure S3**.

| 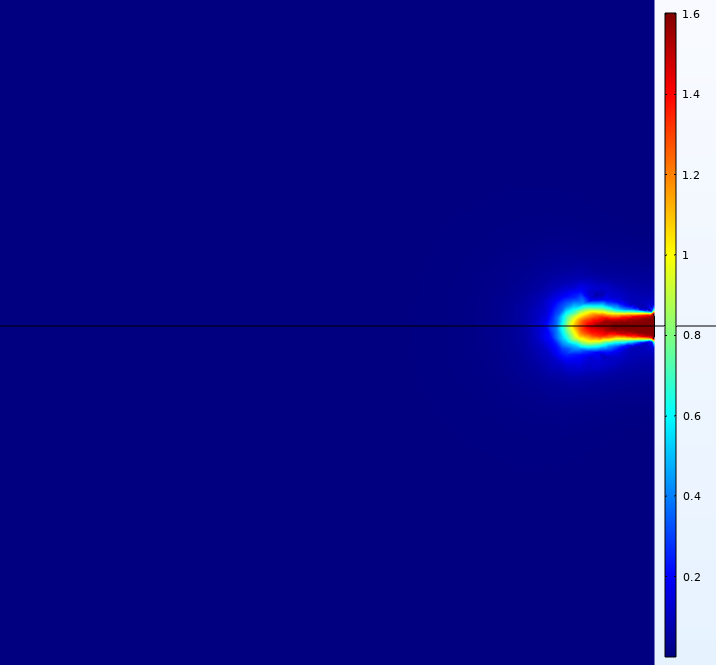 | 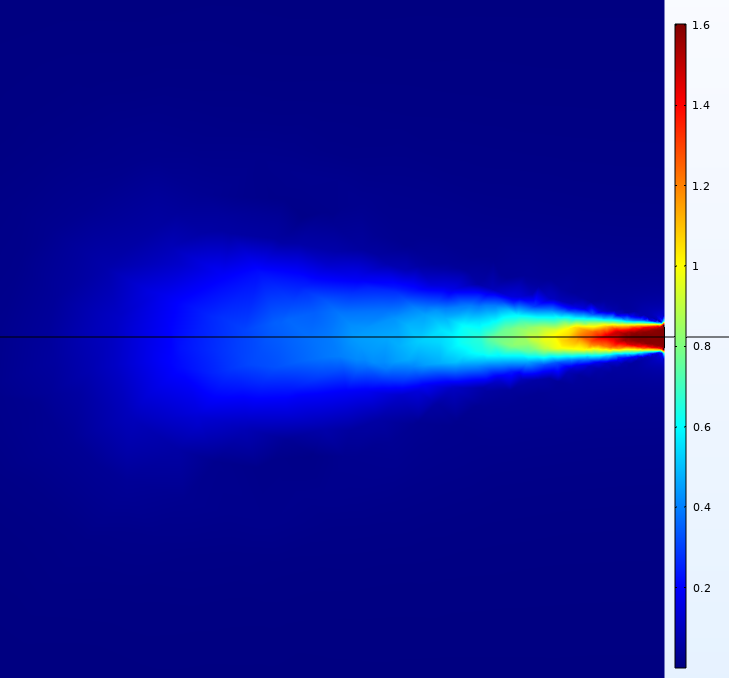 |
| --- | --- |
| 1. T=0.1s | 1. T=1.9s |
| 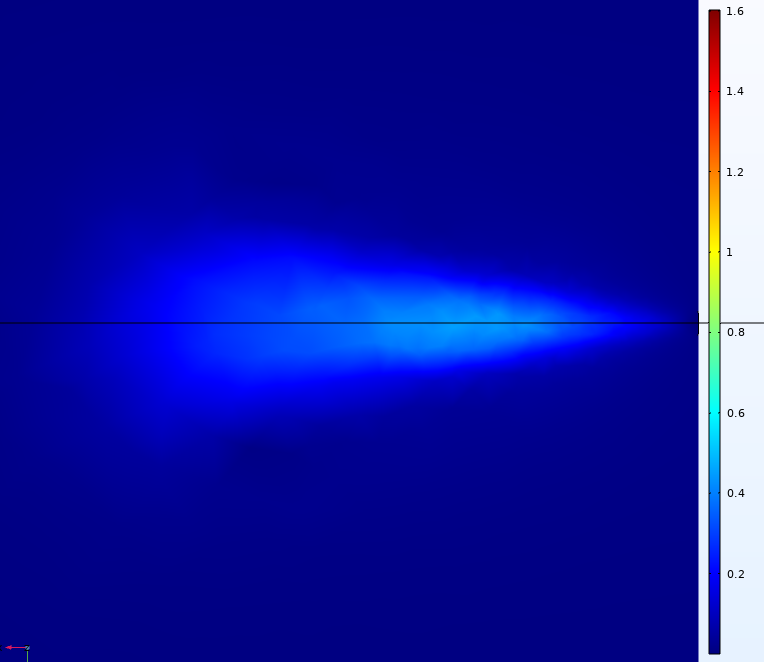 | 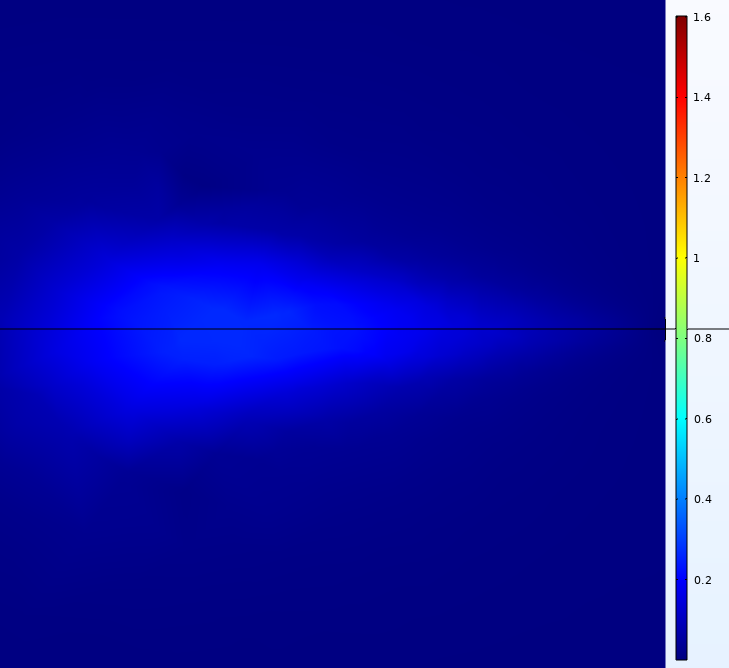 |
| 1. T=2.2s | 1. T=2.6s |
| **Figure S3**: Spatial distribution of the flow velocity from breathing (duration of the emission: 2s) modeled with COMSOL Multiphysics. | |

The simulated centerline velocity (taken as the x component of the flow velocity) is given in **Figure S4** for a sneeze. The velocity is given in m/s.

| 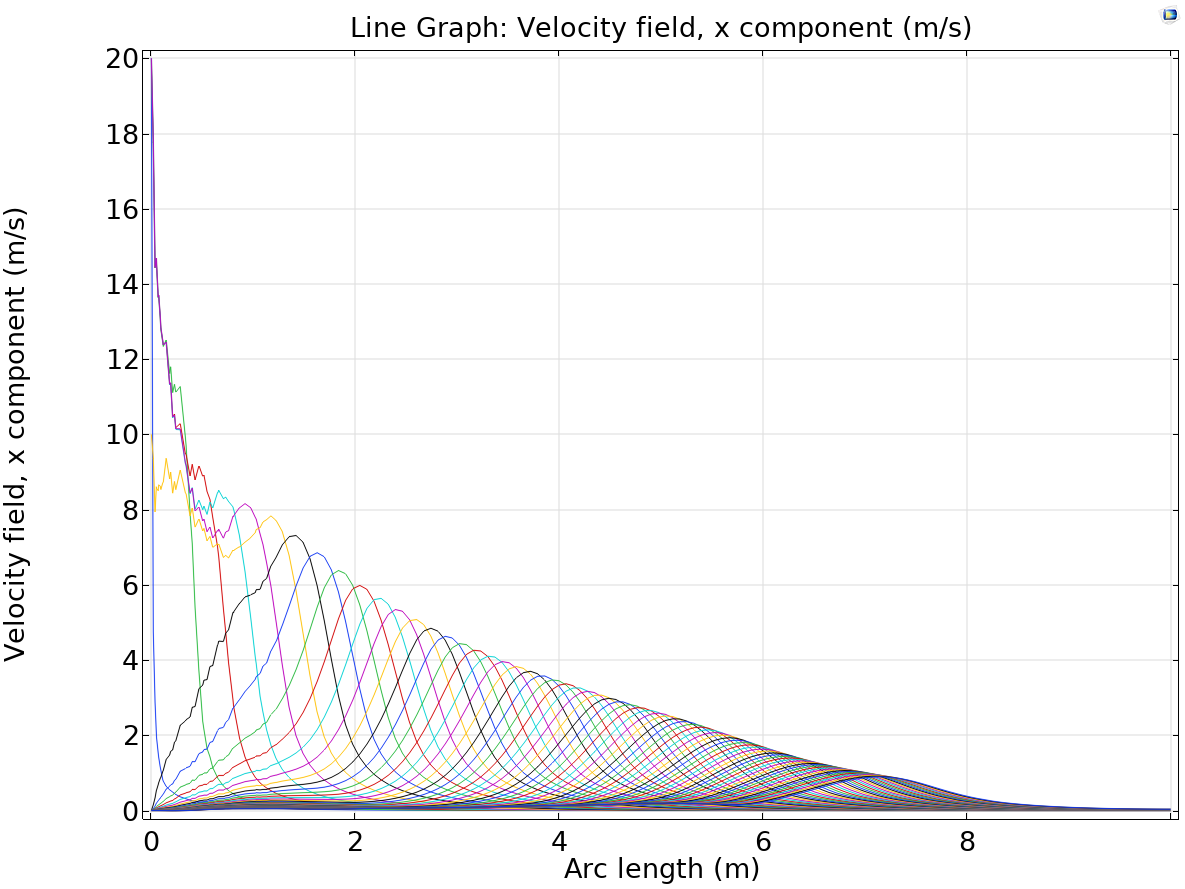 |
| --- |
| **Figure S4**: Centerline velocity profile at different times as a function of the distance from the emission point considering a sneeze. |

The position of the emitted flow was taken as the maximum value of the velocity once the emission stopped, and the centerline velocity was taken at this point to derive the evolution of the velocity as a function of the distance from the emission point. The simulated centerline velocity curve was fitted in Matlab using an exponential function in the form $y=A\cdot e^{-B\cdot x}+C\cdot e^{-C\cdot x}$. The parameters A to C were calculated for the different simulated expiratory activities (breathing, speaking, coughing, and sneezing). The parameters are given in **Table S1**.

|  | A | B | C | D |
| --- | --- | --- | --- | --- |
| Breath | 1.085 | -2.629 | 0.5156 | -25.09 |
| Speak | 2.092 | -16.39 | 1.827 | -1.228 |
| Cough | 6.645 | -1.632 | 5.071 | -18.66 |
| Sneeze | 10.19 | -1.301 | 9.867 | -14.17 |
| **Table S1**: Interpolation parameters for the calculation of the centerline velocity | | | | |

The resulting equations were implemented into the model to update the calculation of the centerline velocity. A comparison of the transient model (adopted in the infection risk model), the original jet model (used in the previous publication [3]) and the steady-state model (CFD verification of the jet model) are given in **Figure S5**.

| 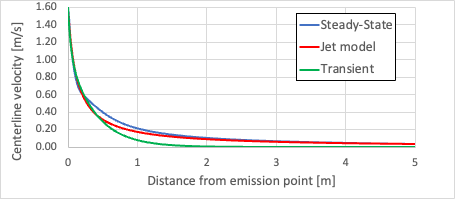 |
| --- |
| 1. Centerline velocity for breathing |
|  |
| 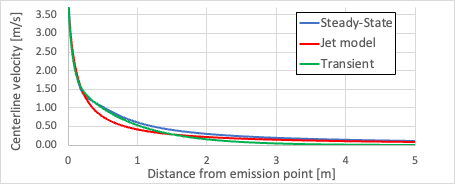 |
| 1. Centerline velocity for speaking |
|  |
| 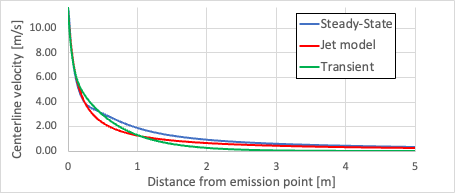 |
| 1. Centerline velocity for coughing |
|  |
| 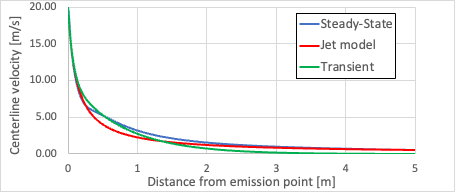 |
| 1. Centerline velocity for sneezing |
| **Figure S5**: Comparison of the calculation of the centerline velocity using the turbulent round jet model, the steady-state CFD model, and the transient CFD model. |

C.4. Initial distributions

Initial distributions were created to describe the variability in the total number of emitted droplets, in the infectivity (dose-relationship parameter), and in the concentration of viral charges in the exhaled droplets.

C.4.1. Viral load of the exhaled droplets

The viral charge concentration values available in the literature have mostly been measured from RT-PCR samples, taken from swabs. The concentration depends on numerous parameters such as the age of the tested individual or the time since infection/apparition of the symptoms. In the present study, we considered the viral charge of an asymptomatic infected individual. The distribution was based on the peak-viral load estimated by Jones et al. [6] from PAMS (pre-symptomatic, asymptomatic and mildly-symptomatic) non-B.1.1.7 (the impact of variants was applied separately on the average of the distribution) subjects. The evolution of the viral load as a function of the time was estimated by Jones et al. [6] to follow a triangular function with a maximum corresponding to the peak viral load, estimated at 7.28 log_10_. The increasing slope was estimated at 2.24 and the decreasing slope at -0.173, allowing a reconstruction of the evolution of the viral charge over time. The duration of the infectious period among asymptomatic individuals has been estimated to start around 3 days before the viral RNA peak concentration and to end 7.8 days after the peak [7]. Other estimation based on symptomatic individuals [6] give the infectious period from 3 days before symptoms onset to 10 days after symptoms onset. In a literature review from Byrne et al. [8], different studies show durations of 11 days from infection to negative test, 9.5 days median duration from diagnosis to negative test (however, the time between infection and diagnosis is unclear), and 4 days for asymptomatic individuals. Considering the high dispersion of these values, we took a conservative approach and considered a total duration of 14 days, based on the recommendations from the CDC [9]. We calculated the average median viral charge between 3 days before the viral charge peak to 10 days after the peak. It was modeled by a log-normal distribution with a median of 5.52 log_10_ and a standard distribution of 1.83 according to data from Jones et al. [6], and represented the average viral charge of an asymptomatic emitter during the contagion time window. The data reported were based on the full dataset and contained non-B.1.1.7 as well as B.1.1.7 patients. However, only 1’533 out of 25’381 positive test results were tested positive for B.1.1.7 and the calculated viral charge was considered to be valid for non-B.1.1.7 individuals. The values were given in copies per swab, Jones et al. [6] reported a sample volume between 2 and 4.3 mL. We considered an average value of 3 mL per sample for the calculations, leading to a **median** concentration of **5.04 log_10_** copies/mL and a **standard deviation** of **1.35 log_10_**. According to Zuin et al. [10], the viral charge concentration was similar in symptomatic and in asymptomatic COVID-19 patients. Based on the data from Watanabe et al. [11] estimating that 300 viral copies were required for a plaque forming unit (PFU), the average viable viral concentration considered in the present work was estimated at **2.56 log_10_** PFU/mL, which corresponds to data presented by Johnson et al. [12]. Our model is based on the viral load given in copies/mL and the conversion into PFU/mL is included in the calculation of the infectivity, see section C.4.3 of the present document for more details.

The viral charge discussed in the previous paragraph was measured from RT-PCR tests and corresponded to the value for a swab, thus taken from the upper respiratory tract, while it has been shown that the viral charge is higher in the lower respiratory tract. A meta-analysis [13] reported the highest positivity rates for PCR tests conducted with sputum samples compared to oropharyngeal and nasopharyngeal swabs. A comparison of 52 patients [14] also reported a higher positivity rate (76.9%) for sputum samples than for throat samples (44.2%). Liu et al. [15] further reported lower cycles counts for RT-PCR tests conducted on sputum samples compared to nasopharyngeal swabs; the time required for a patient to test negative after infection was also higher if the measurement was done with a sputum sample. The differences in viral charge concentration have been estimated in several publications: Wölfel et al. [16] estimated the viral charge in sputum at 7x10^6^ copies per mL (6.85 log_10_) and the average viral load in swabs was 6.76x10^5^ copies per swab. Considering a volume of 3 mL per swab, the equivalent concentration is 2.25x10^5^ copies per mL (5.35 log_10_). The difference between the sputum and the swab was 1.5 log_10_. Fajnzylber et al. [17] reported the viral load in copies/mL of nasopharyngeal, oropharyngeal, sputum, plasma, and urine samples from hospitalized patients. Among all the positive samples, the average viral concentration was 3.88 log_10_ in the nasopharyngeal samples, 3.25 log_10_ in the oropharyngeal samples, and 4.38 log_10_ in the sputum, measured on respectively 32, 31, and 34 patients. A weak correlation was reported for nasopharyngeal vs sputum (r=0.39) and oropharyngeal vs sputum (r=0.56). The difference between swab and sputum was 0.82 log_10_. The reliability of the sputum samples can be discussed, as only a limited fraction of the patients was able to produce sputum, it might not have been representative of asymptomatic patients, as they would have been be less likely to produce sputum samples. Pan et al. [18] compared the viral charge in throat swabs and in sputum. Measurements performed on two patients showed higher viral load in sputum (0.5 log_10_ difference for patient A and 1 log_10_ difference for patient B). Further measurements on 80 patients at different stages of the infection showed an average viral load in the swabs of 5.43 log_10_ and in the sputum of 6.40log_10_. Sun et al. [19] investigated the kinetic of SARS-CoV-2 infections by measuring the viral concentration in nasopharyngeal and throat swabs, and in sputum samples. The viral load was higher in sputum samples compared to the swabs. The peak values were around 10^8^ in sputum, 5x10^6^ in throat swabs and in nasopharyngeal swabs. Based on the data, we attributed a higher viral concentration to the droplets originating from the lower respiratory tract. We chose to consider an average value and implement a **10 times higher** concentration of viral charges in the lower respiratory tract compared to the upper respiratory tract.

There was not consensus on differences between nasopharyngeal and oropharyngeal swabs: Fajnzylber et al. [17] reports a higher viral concentration in nasopharyngeal swab than for oropharyngeal swabs. Mohammadi et al. [13] reported a higher rate of positivity for nasopharyngeal swab than for oropharyngeal swabs. However, the differences were reduced compared to sputum, and Sohn et al. [20] reported similar RT-PCR cycle threshold values in both cases.

Therefore, only differences between the lower and the upper respiratory tract were included in the present study. According to the B.L.O model [21] used to generate the emission size distributions for breathing, speaking, and coughing, the B mode was attributed to the lower respiratory tract, the L and O modes were attributed to the upper respiratory tract. Droplet from a sneeze were considered to be generated in the upper respiratory tract.

C.4.2. Initial emission number

Measurements available in the literature showed a high inter- and intrapersonal variability in the total number of emitted droplets. The emission numbers distribution for the four expiratory activities was compiled from measurements available in the literature (Johnson and Morawska [22], Alsved et al. [23], Duguid [24], Loudon and Roberts [25], Chao et al. [26], Schijven et al. [27], Asadi et al. [28], Morawska et al. [29], Fabian et al. [30], Gerone et al. [31]), the median value and the standard deviation were derived from the data and modeled into a lognormal distribution.

C.4.3. Infectivity parameter

The dose-response relationship models the infection risk as a function of the inhaled viral dose [11]. The infectivity parameter k was estimated by Zhang et al. [32] and considered to follow a triangular distribution with lower limit, upper limit, and mode of 6.4x10^4^, 9.8x10^5^, and 1.6x10^5^ viral copies. We integrated the triangular distribution into the model. The infectivity parameter considers the viability of the virus, based on data for the SARS-CoV Watanabe et al. [11]. Watanabe et al. estimated the dose-response parameter at 400 plaque-forming units, considering that a plaque-forming unit corresponds to 300 viral copies.

C.4.4. Summary


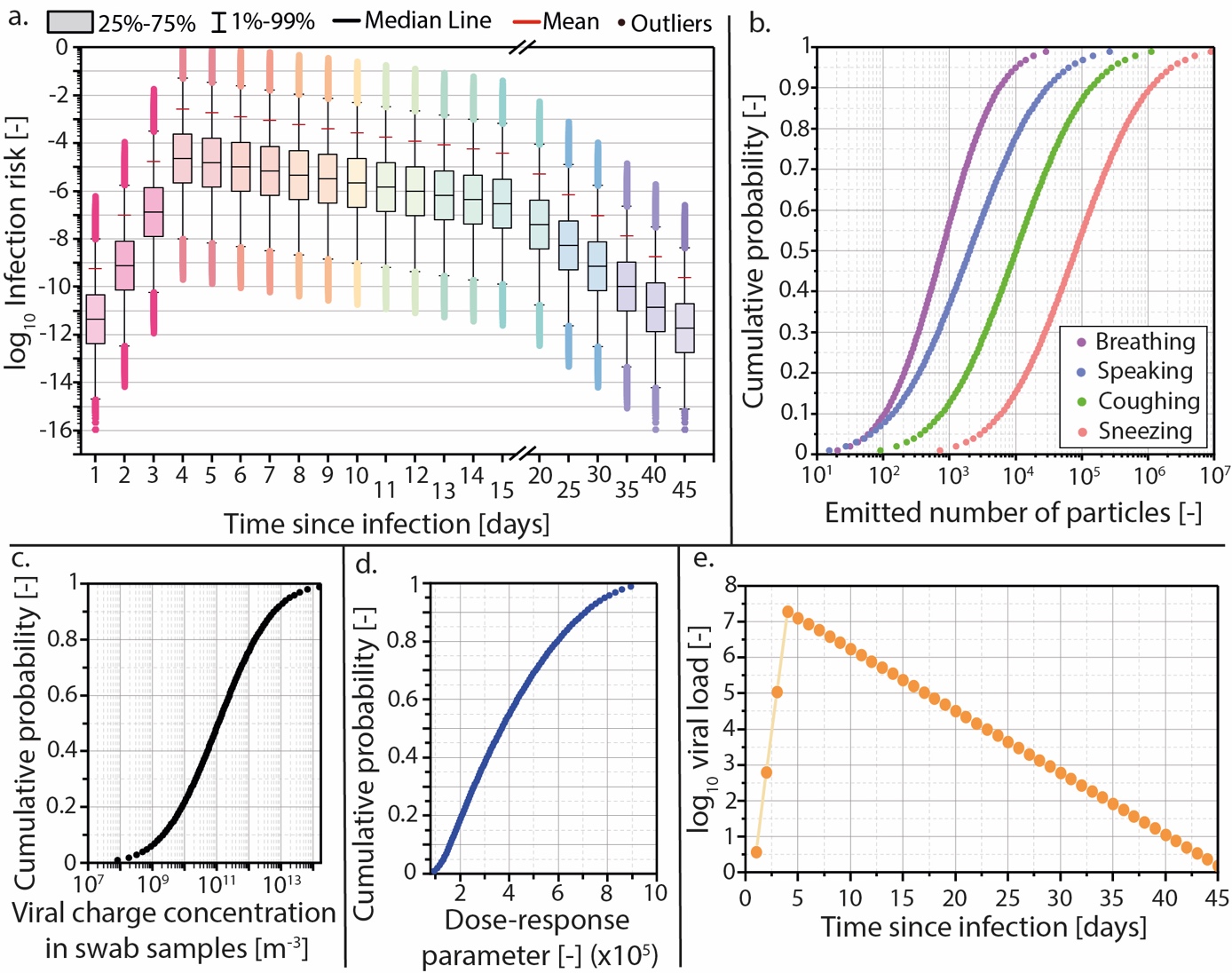


**Figure S6**: Summary of the initial parameters. The evolution of the infection risk distribution in the *Indoor* scenario as a function of the days since infection is given in (a). The total number of emitted particles for the four expiratory activities (breathing, speaking, coughing, and sneezing) is given in (b). The variability of the viral charge concentration in swab samples is given in (c). The uncertainty over the infectivity in the dose-response relationship is given in (d). The evolution of the viral load as a function of the time since infection is given in (e).

1. **Source control versus respiratory protection in the *Office* scenario**

The infection risk in the Office scenario was slightly higher when the masks were worn by the emitter than when the masks were worn by the receiver, while this relation was inverted in the other scenarios (**Figure 3c**). Using a mask for source control has two advantages: blocking large particles before they can reach the receiver, and slowing down the forward oriented airflow, thus reducing the forward motion of the cloud of particles escaping through the mask and the leaks. The Office scenario significantly undermined the advantages of source control as it featured a high interaction time (8 hours) over a long distance (5 m). The distance prevented large particles (>30 μm, diameter at emission) from reaching the receiver even without a mask, as they settled on the floor rapidly (comparatively, particles up to 300 μm can reach the receiver without a mask in the Indoor scenario as the distance is much shorter). The protection from these large particles provided by source control was therefore inconsequential in the Office scenario, as it was already provided by the distance (5 m versus 1 m in the Indoor scenario). Smaller particles were slowed down by the mask and diverted from the forward direction by the leaks but they still reached the receiver in the Office scenario as they were given more time (8 hours) to spread and could travel longer distances than in the Indoor scenario, where the interaction time was much shorter (15 min). The Office scenario therefore constituted the worst-case scenario for the use of masks as source control. A similar effect was measured by Lindsley, et al. [33] for two breathing manikins located 1.8 m away: source control provided the highest short-term protection (<7 minutes), but the long-term protection degraded, and after 15 min respiratory protection became more efficient than source control.

1. **Modeling the impact of vaccination and variants**

The impact of vaccines and variants were estimated from data available in the literature. Both factors impacted the infectivity parameter in the dose-response relationship, and the viral charge concentration. Variants also lead to a modification of the parameters implemented for vaccination, and reflecting the reduction of the protection offered by vaccines for individuals exposed to the variant.

E.1. Vaccination

The influence of vaccination and variants on the infection risk was considered to impact the infectivity of the virus and the emitted viral charge.

Vaccination was found to reduce the infection risk according to phase 3 clinical trials of several vaccines approved by the World Health Organization [34]. The phase 3 clinical trial of the vaccine BNT162b2 [35] showed a 95% reduction in Covid-19 infections (8/21720) compared to the placebo group (162/21728) for individuals 16 years of age or older. The AZD1222 (ChAdOx1 nCoV-19) vaccine showed an efficiency of 62.1% in reducing the infections [36]. The Ad26.COV2.S [37] showed an efficacy of 66.9% in reducing moderate to severe Covid-19 cases and 76.7% in reducing the severe-critical Covid-19 cases after 14 days (85.4% after 28 days). The mRNA-1273 SARS-CoV-2 vaccine showed an efficacy of 94.1% in symptomatic Covid-19 cases compared to the placebo group. Severe cases (30/15210) occurred only in the placebo group [38].

Reductions of the viral charge has also been measured among vaccinated individuals. The vaccine effectiveness was assessed by Pritchard et al. [39] based on RT-PCR positivity, symptoms, and cycle threshold as a proxy for the viral charge. Ct values increased with both increasing time from the first vaccination and increasing number of doses. The highest Ct values were reported among those having received two doses of vaccine or previously/antibody positive. The percentage of positive PCR tests remained stable over the first 20 days following vaccination and decreased after 21 days. Vaccination was associated with a greater reduction in infections with a Ct<30 (high viral load) compared to infection with Ct>30. The vaccination effectiveness (post second dose) against infection with a Ct<30 was estimated at 91%. Vaccination (post second dose) was 95% effective at preventing positive tests results with self-reported symptoms and 58% effective against asymptomatic infections.

The mean Ct value increased from 28.4 (IQR 20.1-32.9) without vaccination or prior infection to 32.7 (IQR 27.7-34) post second dose. The highest Ct values were found among individuals not vaccinated but previously positive >4 months ago (33.2, IQR 31-33.9). Estimations based on the average Ct value among the general population correlated with the vaccination rate give an increase of the Ct values for positively tested vaccinated individuals between 1.61 and 2.61, corresponding to a reduction of the viral load by a factor 1.6-20 [40]. The average Ct value was found to significantly increase in vaccinated individuals 12 days after the first vaccine dose [41]. The reported increase of the Ct values corresponded to a decrease of the viral load in vaccinated individuals by a factor 2.8-4.5. (Shrotri et al. [42]) reported an increase of the mean PCR Ct values 28 days after vaccination from 26.6 before vaccination to 31.3 after vaccination. McEllistrem et al. [43] compared the viral loads of vaccinated and non-vaccinated nursing-home residents with asymptomatic COVID-19. The viral load was significantly higher in non-vaccinated residents than in vaccinated residents. The mean log_10_ viral load dropped from 9.5 for non-vaccinated residents to 7.1 in vaccinated residents, corresponding to mean log_10_ reduction of 2.4.

The clinical trials evaluated the protection provided by vaccines to a receiver facing an infected receiver. Regarding the low vaccination coverage during the clinical trials of the vaccines, it appeared reasonable to consider that the positive cases reported were due to non-vaccinated emitters only. The decrease of the infection rate (e.g. 95%) observed among vaccinated receivers was modelled by a shift in the dose-response relationship corresponding to an infection risk reduced by 95% at equal inhaled viral charge compared to a non-vaccinated receiver.

Data from individuals with a positive PCR test result showed an increase of the cycle threshold linked to a decrease of the viral charge. This was modelled by the reduced concentration of viral charges in the droplets emitted by a vaccinated emitter compared to a non-vaccinated emitter. The literature gave different values for the reduction of the viral charge, we considered an intermediate protection (factor 20).

E.2. Delta variant

The influence of variants on the infection risk was modeled by modifying three parameters: the concentration of viral charges in the emitted droplets, the dose-response parameter, and a multiplier on the vaccination modifiers described earlier modeling a change in the efficiency of vaccination. The parameters for the B.1.617.2 lineage (also known as the Delta variant) were estimated as well as hypothetical future variants that might appear in the future. The vaccine efficiency was lower against the Delta variant: Bernal et al. [44] reports an effectiveness of 93.7% after two doses of the BNT162b2 vaccine and 74.5% for the ChAdOx1 nCoV-19 vaccine with the alpha variant, which dropped to respectively 88% and 67% with the Delta variant, corresponding to an average drop of 8% of the vaccine efficiency. Only the values after two doses were considered. Recent research work from Pouwels et al. [45] and Riemersma et al. [46] suggests that the viral charge was identical in vaccinated and non-vaccinated individuals infected with the Delta variant.

PCR testing of subjects infected with the delta variant revealed a viral charge on average 1’000 times higher compared to the A/B lineage infections during the initial epidemic wave in China in early 2020 [47]. A recent report from the Korean Disease Control and Prevention Agency [48] indicated an average viral load (calculated over 14 days from the infection) 90 times higher for the Delta variant compared to the values measured during the first wave. The peak viral load was reached about 2 days earlier with the delta variant and the viral charge decreased within 10 days to values similar to the other variants. Estimations of the infectivity of the B1.617.2 (Delta variant) compared to the B.1.1.7 (alpha variant) in the UK [49] reported a reproduction number on average 1.7 times higher for the B.1.617.2 variant.

E.3. Omicron variant

Data provided by the UK Health Security Agency showed a drop of the average cycle threshold for PCR tests evaluated in December 2021 compared to the previous months: the average cycle threshold dropped from an average around 30 down to 23 together with the increasing fraction of new infections being linked to the Omicron variant. Riediker et al. [50] extrapolated the data and estimated the average viral load in individuals infected with the Omicron variant to be 10 to 100 times higher compared to the Delta variant. We took an intermediate approach in our model and considered a multiplication of the viral charge by a factor 50 compared to the Delta variant. Riediker et al. [50] also estimated the critical dose to trigger an infection to be 500 virus copies for the wild type SARS-CoV-2, around 300 virus copies for Delta and around 100 copies for Omicron, that we translated into our model by a 3-fold increase of the infectivity (equivalent to a division by 3 of the dose-response parameter in the dose-response relationship).

The Omicron variant was found by early reports to reduce the effectiveness of vaccines compared to previous variants. Khoury et al. [51] estimated that 6 months after the first dose of an mRNA vaccine, the efficacy against Omicron dropped to 40% against symptomatic and 80% against severe disease. A booster shot was estimated to increase the protection, to 86.2% against symptomatic infection and to 98.2% against severe infection. Collie et al. [52] estimated the vaccine effectiveness to be 70% for the Omicron variant compared to 93% for the Delta variant. We included the data from Collie et al. [52] in our model to estimate the protection provided by vaccination.

**Literature for Supporting Information**

**[1]**: Pan, J., et al., Inward and outward effectiveness of cloth masks, a surgical mask, and a face shield, Aerosol Science and Technology, 55, 6, 718-733, 2021, <https://doi.org/10.1080/02786826.2021.1890687>

**[2]**: Mansour, M. M. & Smaldone, G. C., Respiratory Source Control Versus Receiver Protection: Impact of Facemask Fit, Journal of Aerosol Medicine and Pulmonary Drug Delivery, 26, 3, 131-137, 2013, <https://doi.org/10.1089/jamp.2012.0998>

**[3]**: Schmitt J., & Wang, J., Quantitative modeling of the impact of facemasks and associated leakage on the airborne transmission of SARS‑CoV‑2, Sci. Rep., 11, 19403, 2021, | <https://doi.org/10.1038/s41598-021-98895-9>

**[4]**: Oberg, T. & L. M. Brosseau, Surgical mask filter and fit performance, American Journal of Infection Control, 36, 4, 272-282, 2008, <https://doi.org/10.1016/j.ajic.2007.07.008>

**[5]**: Drewnick, F., Pikmann, J., Fachinger, F., Moormann, L., Sprang, F. & Borrmann, S., Aerosol filtration efficiency of household materials for homemade face masks: Influence of material properties, particle size, particle electrical charge, face velocity, and leaks. Aerosol Science and Technology, 55, 1 (2021). <https://doi.org/10.1080/02786826.2020.1817846>

**[6]**: Jones, T.C., et al., Estimating infectiousness throughout SARS-CoV-2 infection course. Science, 373, 6551, 2021. <https://doi.org/10.1126/science.abi5273>

**[7]**: Kissler, S.M., et al., Viral dynamics of acute SARS-CoV-2 infection and applications to diagnostic and public health strategies, PLoS Biol, 19, 7, e3001333, 2021. [https://doi.org/10.1371/journal. pbio.3001333](https://doi.org/10.1371/journal.%20pbio.3001333)

**[8]**: Byrne, A.W., et al., Inferred duration of infectious period of SARS- CoV-2: rapid scoping review and analysis of available evidence for asymptomatic and symptomatic COVID-19 cases, BMJ Open, 10, 0039586, 2020. <https://doi.org/10.1136/bmjopen-2020-039856>

**[9]**: Science Brief: Options to Reduce Quarantine for Contacts of Persons with SARS-CoV-2 Infection Using Symptom Monitoring and Diagnostic Testing, Centers for Disease Control and Prevention, December, 2^nd^, 2020; Consulted online on January 18^th^, 2022. <https://www.cdc.gov/coronavirus/2019-ncov/more/scientific-brief-options-to-reduce-quarantine.html>

**[10]**: Zuin, M., et al., Viral Load Difference between Symptomatic and Asymptomatic COVID-19 Patients: Systematic Review and Meta-Analysis, Infect. Dis. Rep., 13, 645-653, 2021. <https://doi.org/10.3390/idr13030061>

**[11]**: Watanabe, T., et al., Development of a Dose-Response Model for SARS Coronavirus, Risk Analysis, 30, 7, 2010. <https://doi.org/10.1111/j.1539-6924.2010.01427.x>

**[12]**: Johnson, T.J., et al., Viral load of SARS‑CoV‑2 in droplets and bioaerosols directly captured during breathing, speaking and coughing, Scientific Reports, 12, 3484, 2022. <https://doi.org/10.1038/s41598-022-07301-5>

**[13]**: Mohammadi, A., et al., SARS-CoV-2 detection in different respiratory sites: A systematic review and meta-analysis, EBioMedicine, 59, 102903, 2020. <https://doi.org/10.1016/j.ebiom.2020.102903>

**[14]**: Lin, C., et al., Comparison of throat swabs and sputum specimens for viral nuclei acid detection in 52 cases of novel coronavirus (SARS-Cov-2)-infected pneumonia (COVID-19), Clin Chem Lab Med, 58, 7, 1089-1094, 2020. <https://doi.org/10.1515/cclm-2020-0187>

**[15]**: Liu, R., et al., Viral Load Dynamics in Sputum and Nasopharyngeal Swab in Patients with COVID-19, Journal of Dental Research, 99, 11, 1239-1244, 2020. <https://doi.org/10.1177/0022034520946251>

**[16]**: Wölfel, R., et al., Virological assessment of hospitalized patients with COVID-2019, Nature, 581, 465-469, 2020. <https://doi.org/10.1038/s41586-020-2196-x>

**[17]**: Fajnzylber, J., et al., SARS-CoV-2 viral load is associated with increased disease severity and mortality, Nature Communications, 11, 5493, 2020. <https://doi.org/10.1038/s41467-020-19057-5>

**[18]**: Pan, X., et al., Viral load of SARS-CoV-2 in clinical samples, Lancet Infect Dis., 20, 4, 411-412, 2020. <https://doi.org/10.1016/S1473-3099(20)30113-4>

**[19]**: Sun, J., et al., The kinetics of viral load and antibodies to SARS-CoV-2, Clinical Microbiology and Infection, 26, 2020. <https://doi.org/10.1016/j.cmi.2020.08.043>

**[20]**: Sohn, Y., et al., Assessing Viral Shedding and Infectivity of Asymptomatic or Mildly Symptomatic Patients with COVID-19 in a Later Phase, J. Clin. Med., 9, 2924, 2020. <https://doi.org/10.3390/jcm9092924>

**[21]**: Johnson, G.R., et al., Modality of human expired aerosol size distributions, Journal of Aerosol Science, 42, 839-851, 2011. <https://doi.org/10.1016/j.jaerosci.2011.07.009>

**[22]**: Johnson, G.R. & Morawska, L., The Mechanism of Breath Aerosol Formation, Journal of Aerosol Medicine and Pulmonary Drug Delivery, 22, 3, 2009. <https://doi.org/10.1089/jamp.2008.0720>

**[23]**: Alsved, M., et al., Exhaled respiratory particles during singing and talking, Aerosol Science and Technology, 54, 11, 1245-1248, <https://doi.org/10.1080/02786826.2020.1812502>

**[24]**: Duguid, J. P., ﻿The size and duration of air-carriage of respiratory droplets and droplet-nuclei. *J Hyg (Lond)*, **44,** 6, 471–479 (1946). <https://doi.org/10.1017/s0022172400019288>

**[25]**: Loudon, R. G. & Roberts, R. M., Droplet expulsion from the respiratory tract. *Am Rev Respir Dis*, **95,** 3, 435-42 (1967). <https://doi.org/10.1164/arrd.1967.95.3.435>

**[26]**: Chao, C. Y. H., et al., Characterization of expiration air jets and droplet size distributions immediately at the mouth opening. Aerosol Science, 40, 122 – 133 (2009). <https://doi.org/10.1016/j.jaerosci.2008.10.003>

**[27]**: Schijven, J., *et al.,* Exposure assessment for airborne transmission of SARS-CoV-2 via breathing, speaking, coughing and sneezing. preprint on *medRxiv* (2020). <https://doi.org/n10.1101/2020.07.02.20144832>

**[28]**: Asadi, S., Wexler, A. S., Cappa, C. D., Barreda, S., Bouvier, N. M.& Ristenpart, W. D., Effect of voicing and articulation manner on aerosol particle emission during human speech. PLoS ONE, 15, 1 (2020). [https://doi.org/10.1371/journal. pone.0227699](https://doi.org/10.1371/journal.%20pone.0227699)

**[29]**: ﻿Morawska, L., et al., ﻿Size distribution and sites of origin of droplets expelled from the human respiratory tract during expiratory activities. ﻿*Aerosol Science*, **40,** 256-269 (2009). ﻿<https://doi.org/10.1016/j.jaerosci.2008.11.002>

**[30]**: Fabian, P., Brain, B., Houseman, A. E., Gern, J. & Milton, D. K., Origin of exhaled breath particles from healthy and human rhinovirus-infected subjects. Journal of Aerosol Medicine and Pulmonary Drug Delivery, 24, 3 (2011). <https://doi.org/10.1089/jamp.2010.0815>

**[31]**: Gerone, P. J., Couch, R. B., Keefer, G. V., Douglas, R. G., Derrenbacher, E. B. & Knight, V., ﻿Assessment of experimental and natural viral aerosols. *Bacteriological Reviews*, **30,** 3 (1966). <https://doi.org/10.1128/br.30.3.576-588.1966>

**[32]**: Zhang, X., Ji, Z., Yue, Y., Liu, H. & Wang, J., Infection Risk Assessment of COVID-19 through Aerosol Transmission: a Case Study of South China Seafood Market. *Environ. Sci. Technol.*, **55,** 7, 4123–4133 (2021). <https://doi.org/10.1021/acs.est.0c02895>

**[33]**: Lindsley, W.G., Efficacy of universal masking for source control and personal protection from simulated cough and exhaled aerosols in a room, 2021, 18, 8, 409-422, <https://doi.org/10.1080/15459624.2021.1939879>

**[34]**: COVID19 Vaccine Tracker, World Health Organization. Consulted on January, 18^th^, 2022. <https://covid19.trackvaccines.org/agency/who/>

**[35]**: Polack, F.P., et al., Safety and Efficacy of the BNT162b2 mRNA Covid-19 Vaccine, N Engl J Med, 383, 2603-2615, 2020. <https://doi.org/10.1056/NEJMoa2034577>

**[36]**: Voysey, M., et al., Safety and efficacy of the ChAdOx1 nCoV-19 vaccine (AZD1222) against SARS-CoV-2: an interim analysis of four randomised controlled trials in Brazil, South Africa, and the UK, Lancet, 397, 99-111, 2021. <https://doi.org/10.1016/S0140-6736(20)32661-1>

**[37]**: Sadoff, J., et al., Safety and Efficacy of Single-Dose Ad26.COV2.S Vaccine against Covid-19, N Engl J Med, 384, 2187-2201, 2021. <https://doi.org/10.1056/NEJMoa2101544>

**[38]**: Baden, L.R., et al., Efficacy and Safety of the mRNA-1273 SARS-CoV-2 Vaccine, N Engl J Med, 384, 403-416, 2021. <https://doi.org/10.1056/NEJMoa2035389>

**[39]**: Pritchard, E., et al., Impact of vaccination on new SARS-CoV-2 infections in the United Kingdom, Nature Medicine, 27, 1370-1378, 2021. <https://doi.org/10.1038/s41591-021-01410-w>

**[40]**: Petter, E., et al., Initial real world evidence for lower viral load of individuals who have been vaccinated by BNT162b2, preprint posted on medRxiv on February 8^th^, 2021. <https://doi.org/10.1101/2021.02.08.21251329>

**[41]**: Levine-Tiefenbrun, et al., Initial report of decreased SARS-CoV-2 viral load after inoculation with the BNT162b2 vaccine, Nature Medicine, 27, 790-792, 2021. <https://doi.org/10.1038/s41591-021-01316-7>

**[42]**: Shrotri, M., et al., Vaccine effectiveness of the first dose of ChAdOx1 nCoV-19 and BNT162b2 against SARS-CoV-2 infection in residents of long-term care facilities in England (VIVALDI): a prospective cohort study, Lancet Infect Dis., 21, 11, 1529-1538, 2021. <https://doi.org/10.1016/S1473-3099(21)00289-9>

**[43]**: McEllistrem, M.C., et al., Single dose of a mRNA SARS-CoV-2 vaccine is associated with lower nasopharyngeal viral load among nursing home residents with asymptomatic COVID-19, Clin Infect Dis, 73, 6, e1365-e1367, 2021. <https://doi.org/10.1093/cid/ciab263>

**[44]**: Bernal, J.L., et al., Effectiveness of Covid-19 Vaccines against the B.1.617.2 (Delta) Variant, N Engl J Med, 385, 585-594, 2021. <https://doi.org/10.1056/NEJMoa2108891>

**[45]**: Pouwels, K.B., et al., Effect of Delta variant on viral burden and vaccine effectiveness against new SARS-CoV-2 infections in the UK, Nature Medicine, 27, 2127-2135, 2021. <https://doi.org/10.1038/s41591-021-01548-7>

**[46]**: Riemersma, K.K., et al., Shedding of Infectious SARS-CoV-2 Despite Vaccination, preprint on medRxiv published on November, 6^th^, 2021. <https://doi.org/10.1101/2021.07.31.21261387>

**[47]**: Li, B., et al., Viral infection and transmission in a large, well-traced outbreak caused by the SARS-CoV-2 Delta variant, preprint published on medRxiv on July, 23^rd^, 2021. <https://doi.org/10.1101/2021.07.07.21260122>

**[48]**: Korean Disease Control and Prevention Agency, Public Health Weekly Report, Vol.14, No.35, 2495, 2021. Published on September 9^th^, 2021.

**[49]**: Dagpunar, J., Interim estimates of increased transmissibility, growth rate, and reproduction number of the Covid-19 B.1.617.2 variant of concern in the United Kingdom, preprint published on medRxiv, on June, 3^rd^, 2021. <https://doi.org/10.1101/2021.06.03.21258293>

**[50]**: Riediker, M., et al., Higher viral load and infectivity increase risk of aerosol transmission for Delta and Omicron variants of SARS-CoV-2, Swiss Med Wkly, 2022. <https://doi.org/10.4414/SMW.2022.w30133>

**[51]**: Khoury, D.S., et al., A meta-analysis of Early Results to predict Vaccine efficacy against Omicron, preprint published on medRxiv on December, 17^th^, 2021. <https://doi.org/10.1101/2021.12.13.21267748>

**[52]**: Collie, S., et al., Effectiveness of BNT162b2 Vaccine against Omicron Variant in South Africa, N Engl J Med, 2021, <https://doi.org/10.1056/NEJMc2119270>
